# Locating Impurity Phases in the Lithium-Ion Conductor Al-Doped Li_7_La_3_Zr_2_O_12_ through Dynamic Nuclear Polarization and Nuclear Magnetic Resonance Spectroscopy

**DOI:** 10.1021/acs.chemmater.5c00807

**Published:** 2025-05-13

**Authors:** Astrid H. Berge, Sundeep Vema, Christopher A. O’Keefe, Clare P. Grey

**Affiliations:** Yusuf Hamied Department of Chemistry, 2152 University of Cambridge, Lensfield Road, Cambridge CB2 1EW, U.K.

## Abstract

An understanding of the nature of the grain boundaries and impurity phases contained in complex mixed metal oxide solid electrolytes is key to the development of improved and more stable solid-state batteries with reduced grain boundary resistances and higher ionic conductivities of the bulk sample. The Li-ion solid electrolyte Li_7_La_3_Zr_2_O_12_ (LLZO) is one of the most researched electrolytes in the field due to its high ionic conductivity, thermal stability, and wide voltage stability window. Despite its potential, the nature of the impurity and surface phases formed during the synthesis of LLZO and their role and influence on LLZO’s performance when used as an electrolyte remain poorly understood and controlled. In addition, there are limited characterization methods available for detailed studies of these impurity phases, particularly if these phases are buried in or close to the grain boundaries of a dense sintered material. Here, we demonstrate a solid-state nuclear magnetic resonance (ssNMR) and dynamic nuclear polarization (DNP) approach that exploits both endogenous and exogenous dopants to select for either specific impurities or separate bulk vs surface/subsurface phases. Specifically, the location of Al-containing phases within an Al doped LLZO and the impurity phases that form during synthesis are mapped: by doping LLZO with trace amounts of paramagnetic metal ions (Fe^3+^ and Gd^3+^), DNP is used to selectively probe Al- and La-containing impurity phases, respectively, allowing us to enhance the signals arising from the LiAlO_2_ and LaAlO_3_ impurities and to confirm their identity. A ^17^O DNP experiment using Gd^3+^ doped LLZO is performed to identify further La^3+^-containing impurities (specifically La_2_Zr_2_O_7_ and La_2_O_3_). Finally, a ^7^Li DNP irradiated ^7^Li–^27^Al dipolar-based heteronuclear multiple quantum correlation experiment is performed by using the radical TEKPol as the polarization agent. This experiment demonstrates that the poorly crystalline LiAlO_2_ that is found close to the surfaces of the LLZO composite is coated by a thin Li-containing impurity layer and thus not directly present at the surface.

## Graphical abstract

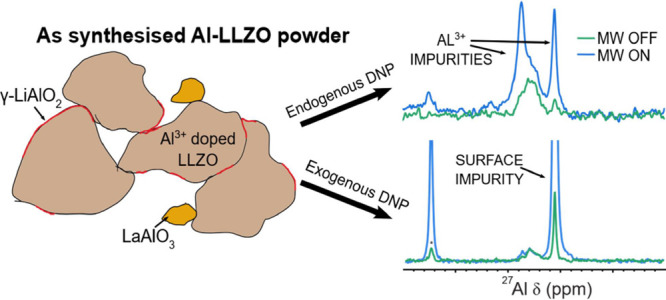

## Introduction

Current battery research focuses on improving and overcoming the remaining limitations facing batteries, namely increasing their longevity, energy density, and safety while ensuring that the source materials are environmentally friendly. One strategy, moving forward, is to substitute the lithium-ion-conducting liquid electrolyte with a solid electrolyte. Solid-electrolyte-based lithium-ion batteries can, in principle, enable energy storage devices with high energy and power densities due to the compatibility of some solid electrolytes with high voltage cathodes (>4.5 V vs Li/Li^+^) and the promise that Li metal can be used as an anode; critically, they are also less flammable and more thermally stable than batteries with liquid electrolytes.^1,2^ While the challenges associated with the optimization of these batteries are large, the first challenge involves an optimization of the ionic conductivity of the bulk and at the grain boundaries and interfaces involving the solid-state electrolyte. It is not sufficient to simply understand the structure of solid electrolyte particles in order to rationalize their performance, but the nature of the grain boundaries and any impurity phases formed on synthesis and on densification of the electrode needs to be understood and controlled in order to optimize performance.

Since it was first synthesized in 2007,^3^ the Li-ion solid electrolyte Li_7_La_3_Zr_2_O_12_ (LLZO) has become one of the most researched electrolytes in the field due to its high ionic conductivity, thermal stability, and wide voltage stability window. It is also one of the few electrolytes that are stable against Li metal.^4^ Undoped LLZO has a tetragonal crystal structure (*I*41/*acd* space group), which upon doping with a sufficiently high amount of a multivalent cation, becomes cubic ( $Ia\overset{-}{3}d$ ) as the dopant replaces Li^+^, La^3+^ or Zr^4+^ in the structure.^5^ The cubic lattice has higher Li^+^-ion connectivity, creating a network of channels for Li^+^ diffusion and a high Li-ion conductivity on the order of 10^–4^ S cm^–1^.^6^ The exact conductivity of LLZO depends upon the dopant atom (Al, Ga, Ta, etc.), dopant concentration, synthesis, and processing conditions.

Recent work done by our group has shown that Al^3+^ dopes in the tetragonal 24d Li^+^ site within LLZO.^7^ In addition, Al^3+^ doping also results in the simultaneous formation of the Al^3+^-containing impurities γ-LiAlO_2_ and LaAlO_3_, when using typical synthesis methods involving excess Li^+^ (added to compensate for Li evaporation); these impurities each consume approximately 15–30% of the added Al dopant, as quantified by nuclear magnetic resonance (NMR) spectroscopy. Thus, the stoichiometry of Al^3+^ in synthesized LLZO differs significantly from the intended concentration, affecting the resulting conductivity of the solid electrolyte. The formation of LaAlO_3_ was seen by both powder X-ray diffraction (PXRD) and NMR, while the LiAlO_2_ could only be detected by NMR. The analogous LiGaO_2_ impurity was formed during the synthesis of Ga doped LLZO and was again not observed by PXRD. The levels of the LiAlO_2_ impurity were then controlled by reducing the Li excess, resulting in higher Al-incorporation into the LLZO phase, with a reduction in grain boundary resistance (LiAlO_2_ is the major contributor to grain boundary resistance due to its low ionic conductivity (<10–15 mS cm^–1^) compared to LLZO), but with a concomitant reduction in bulk Li transport; the latter was ascribed to the reduction of Li ions (charge carriers) in the structure and more Al^3+^ ions present that block conduction pathways.^8^ These observations motivated a more extensive study of the impurity phases in LLZO and the development of approaches to investigate whether any of these impurity phases are located on the surface of the LLZO particles or in the grain boundaries of the LLZO dense ceramic.

One method exploited in this work is Dynamic Nuclear Polarization (DNP) NMR spectroscopy. This is a growing field within solid-state NMR, opening the possibility of studying low-abundance species and environments by significantly enhancing their NMR signal^9,10^ or obtaining structural information by selective polarization.^11–13^ This is achieved by transferring the large electron polarization of a nearby unpaired electron to the NMR nuclei of interest. This can be performed via three mechanisms, namely, the Overhauser effect, the solid effect, and the cross effect. For metal ions, the solid effect is thought to dominate.^14^ DNP has been shown to work with several polarization sources, ranging from organic radicals to paramagnetic ions and lithium metal. Within the battery community, this has opened the possibility of studying the interfaces between the electrodes and the electrolyte as well as selectively investigating phases of interest within the battery system^13,15–18^ Here, we use the DNP method to not only enhance the signal of specific phases but also help identify their location within the complex LLZO composite oxide, this being composed of the desired phase and a series of impurity and grain boundary/intergrowth/surface phases.

In DNP, the radical causing the DNP enhancement can either be part of the sample itself (endogenous) or added to the sample afterward (exogenous). The most common endogenous metal ion sources are those with no orbital angular momentum (*L*) and thus no spin orbit coupling (e.g., Gd^3+^, Fe^3+^, and Mn^2+^). This results in the paramagnetic ions having a g-factor close to that of the free electron and a longer electron spin–lattice relaxation time (*T*_1e_), making them suitable for metal-ion DNP.^19,20^ In a recent and elegant example of the approach, Steinberg et al. used the combination of endogenous (Mn^2+^-ions substituted into the oxide Li_4_Ti_5_O_12_, LTO) and exogenous radicals (TEKPol nitroxide biradicals^21^ sorbed on the surface) to identify phases in the inner and outer regions of the solid electrolyte interphase (SEI) formed on the LTO particles in a sodium-ion battery.^18^

The DNP experiment can be performed as a direct experiment in which the nucleus of interest is polarized directly through its hyperfine interaction with the radical. For this to then result in a DNP enhancement of a larger fraction of the bulk, the probed nuclei must be connected to each other via sufficiently rapid spin diffusion. The extent of spin diffusion is determined by the homonuclear dipolar interaction, which is stronger for nuclei with high gyromagnetic ratios and decreases with the cube of the distance between the nuclei. An alternative to direct DNP is indirect DNP, where a nucleus with a high gyromagnetic ratio and natural abundance (usually protons) and, hence, good spin diffusion is polarized by the radical. This polarization is then transferred to the nuclei of interest through a subsequent polarization transfer step, for example, cross-polarization (CP).

Recent progress in developing better polarization transfer steps between quadrupolar nuclei under magic angle spinning (MAS) has yielded sequences such as dipolar-based heteronuclear multiple quantum correlation (D-HMQC).^22,23^ This sequence can offer more efficient polarization transfer for quadrupolar nuclei than CP, circumventing problems relating to normal Hartman-Hahn CP, such as difficulties in spin locking due to short spin-lock relaxation times (*T*_1ρ_). Previously, D-HMQC sequences have been used to probe dipolar interactions between pairs of quadrupolar nuclei, including ^11^B–^17^O,^24,25^ ^11^B–^27^Al,^26^ and ^23^Na–^27^Al.^27^

In this article, the Al-substituted LLZO system, including its impurity phases, is investigated by selective doping using either endogenous L = 0 radicals (Gd^3+^ and Fe^3+^) or the exogenous biradical TEKPol.^21^ The dopants Gd^3+^ and Fe^3+^ were chosen based on their size and charge, as they are expected to selectively dope for La^3+^ and Al^3+^, respectively (Table S1). Therefore, Gd^3+^ was expected to dope into the LaAlO_3_ and LLZO phases, while Fe^3+^ should, in principle, substitute into LaAlO_3_, LLZO, and γ-LiAlO_2_, allowing the LaAlO_3_ and LiAlO_2_ phases to be distinguished on the basis of different relative enhancements. As shown in our previous publication,^7^ the Al^3+^ ions in LLZO are very dilute. This means that the ^27^Al spins are not connected via strong ^27^Al–^27^Al dipolar interactions. Thus, very limited, if any, ^27^Al–^27^Al spin diffusion results. We show, in this work, that this means that in a direct ^27^Al DNP experiment, Al-LLZO cannot be well enhanced, allowing selective enhancement of the Al-containing impurities, LiAlO_2_ and LaAlO_3_, formed alongside the LLZO. Finally, a direct ^27^Al DNP experiment polarized by TEKPol is compared to a ^7^Li–^27^Al D-HMQC experiment polarized by TEKPol to determine which phases are present on the surface of LLZO.

## Experimental Section

### Synthesis of Al-LLZO Powders

Al-LLZO (Al_0.36_Li_5.92_La_3_Zr_2_O_12_) was synthesized using a solid-state method with 10% excess Li in MgO crucibles to avoid unintentional Al doping as described in detail in a previous study.^28^ The calcination temperature was 1000 °C. For Gd-doped Al-LLZO, 0.1 mol % La_2_O_3_ was substituted for Gd_2_O_3_. The Fe-doped LLZO was made with 0.1 mol % Fe_2_O_3_ substituted 1:1 for Li_2_CO_3_. All synthesized LLZO powders were transferred above 150 °C to a glovebox to prevent any reaction with moisture.

Acetone (HPLC-grade) was dried over molecular sieves (3 Å) overnight. TEKPol-doped LLZO was prepared by mixing 1 wt % TEKPol with Al-LLZO in acetone inside a glovebox. The acetone was evaporated before the material was packed into a rotor. A new sample was made for each measurement day.

### ^17^O Enrichment of Gd^3+^-Doped Al-LLZO

A 100 mg portion of Gd^3+^ doped Al-LLZO was put into a cylindrical Al_2_O_3_ crucible and heated to 600 °C under 1 bar of 70% enriched ^17^O_2_ gas overnight using a 13 mL quartz crucible. This gives a maximum theoretical enrichment of 1 in 4 of all the oxygens in LLZO.

### Scanning Electron Microscopy

The SEM image was captured using a Tescan MIRA3 FEG-SEM instrument using an acceleration voltage of 10.0 kV. The sample was mounted onto the SEM stage of an air-sensitive transfer module (Kammrath & Weiss, type CT0) inside a glovebox and transferred into the SEM chamber while maintaining an inert argon atmosphere.

### Magic Angle Spinning Nuclear Magnetic Resonance Spectroscopy

The DNP MAS NMR spectra were recorded on a 400 MHz (9.4 T) magnet with an Avance NEO console using a Bruker 1.3 mm HXY probe at 100 K. ^1^H, ^7^Li, ^27^Al, and ^17^O pulses were optimized and chemical shifts were referenced using adamantane (1.9 ppm), LiF (−1 ppm), AlF_3_ (−17 ppm) and H_2_O (0 ppm), respectively. The microwave (MW) source was a Klystron operating at 263 GHz. 5.2 W MW power was used for all measurements.

Dipolar-based heteronuclear multiple quantum correlation (D-HMQC) experiments between ^7^Li and ^27^Al were conducted with an initial rotor-assisted polarization transfer (RAPT) on ^27^Al. The RAPT was optimized on the sample, giving an offset frequency of ± 250 kHz and an rf power of 53 kHz using Gaussian-shaped pulses. The D-HMQC used a rotational-echo double-resonance (REDOR) recoupling sequence of 5 rotor periods applied to ^7^Li. Coherences of the form *I*_*x*_ *S*_*z*_, where *I* = ^27^Al and *S* = ^7^Li, are generated at the end of the REDOR evolution period. A π/2 pulse applied on ^7^Li generates multiple quantum (MQ) coherences of the form *I*_*x*_ *S*_*x*_; the second π/2 pulse and appropriate phase cycling result in the coherence *I*_*x*_ *S*_*z*_, thereby selecting for Al environments close to ^7^Li. LiAlO_2_ was used as a standard sample to optimize the parameters of the D-HMQC sequence.

The 700 MHz (16.4 T) and the 500 MHz (11.7 T) spectra were recorded using an Avance IIIHD console and a 1.3 mm HX probe and a 2.5 mm HX probe, respectively. The ^27^Al experiments were performed using a spin–echo (30°-τ-60°-τ-acquire) pulse sequence. Pulse optimizations and referencing were done on AlF_3_ (−17 ppm). A short pulse (π/24) with a recycle delay of 5 s was used to quantify the different Al species.

## Results

### Synthesis and Characterization of the LLZO Composite

Three batches of Al-LLZO were synthesized with the nominal stoichiometry Al_0.36_Li_5.92_La_3_Zr_2_O_12_. The first was prepared without any paramagnetic dopants, the second with 0.1% of La^3+^ replaced by Gd^3+^, and the third with 0.1% of Li^+^ replaced by Fe^3+^ as detailed in the methods section. These samples are termed Al-LLZO, Gd^3+^-doped Al-LLZO, and Fe^3+^-doped Al-LLZO throughout. In addition, a solution of acetone containing the organic biradical TEKPol was added to the Al-LLZO powder, and the suspension was dried to create an Al-LLZO sample with polarization sites (radicals) on the LLZO surface. Dopant concentrations were chosen based on concentrations used previously in the DNP literature for oxides and surface doping.^29,30^

Synchrotron PXRD patterns for the synthesized Al-LLZO, Gd^3+^-doped Al-LLZO, Fe^3+^-doped Al-LLZO, and TEKPol-doped Al-LLZO were recorded and showed the formation of LLZO with only low concentrations of the impurity phases (LaAlO_3_ and Li_2_ZrO_3_); all the PXRD patterns are similar, indicating that the Gd^3+^ and Fe^3+^ additions do not affect the crystal structure of LLZO at the dopant levels used (Figure S1). More specifically, 1.5, 0.9, and 1.6 wt % of LaAlO_3_ in the Al-LLZO, Gd^3+^-doped Al-LLZO, and Fe^3+^-doped Al-LLZO samples, respectively, were determined by Rietveld refinement using the PXRD data. For Fe^3+^ and undoped LLZO, some tetragonal LLZO was formed alongside the cubic LLZO.

To investigate the effect of the addition of paramagnetic centers on impurity concentrations further, the ^27^Al MAS NMR spectra were measured for each sample (Figure 1), showing the presence of the same three ^27^Al resonances in each of the Al-LLZO samples. These three resonances (with isotopic chemical shifts of 80.1, 68.8, and 11.0 ppm determined by the simultaneous fitting of data acquired at field strengths of 9.4, 16.4, and 23.5 T (Figure S2)) have been assigned to LiAlO_2_, LLZO, and LaAlO_3_, respectively.^7^ The distribution of Al^3+^ between these phases was quantified by NMR (Table 1). This distribution was used to calculate the stoichiometry of LLZO and the phase fraction of LLZO, LaAlO_3_, and LiAlO_2_ in the samples (see SI for full details). Interestingly, the weight fraction of LaAlO_3_ calculated by NMR is higher for all samples than that calculated by PXRD. This is likely due to NMR being able to detect poorly crystalline and amorphous LaAlO_3_.

**Table 1:** Al Distributions between the Three Al-Containing Phases (LLZO, LiAlO_2_, and LaAlO_3_) as Quantified by NMR (11.7 T, ν_rot_ = 20 kHz) and the resulting LLZO Stoichiometries^a^ ^,^ ^b^

	Al^3+^ in LLZO			Al^3+^ in LiAlO_2_			Al^3+^ in LaAlO_3_			
	amount of total Al	molar phase fraction	weight phase fraction	amount of total Al	molar phase fraction	weight phase fraction	amount of total Al	molar phase fraction	weight phase fraction	LLZO stoichiometry
Al-LLZO	36%	77.9%	95.1%	27%	9.3%	0.9%	37%	12.8%	4.0%	Al_0.16_Li_6.52_La_3_Zr_2_O_12_
Gd^3+^-doped Al-LLZO	46%	83.4%	96.1%	16%	4.9%	0.4%	38%	11.7%	3.4%	Al_0.17_Li_6.49_La_3_Zr_2_O_12_
Fe^3+^-doped Al-LLZO	44%	83.1%	97.3%	37%	11.2%	1%	19%	5.7%	1.7%	Al_0.16_Li_6.52_La_3_Zr_2_O_12_

a This Al distribution was used to calculate the phase fraction of the three species. This was done both as a molar phase fraction and a weight phase fraction. Note that molar phase fractions are calculated per formula unit, i.e., Al-LLZO contains approximately 80% Al_0.16_Li_6.52_La_3_Zr_2_O_12_, 9% LiAlO_2_, and 5% LaAlO_3_. A full explanation of the calculations can be found in the SI. The ^27^Al NMR spectra were acquired using a short pulse length (π/24) and a long relaxation delay (5 s) to ensure that quantitative spectra were obtained.

b The % distribution of Al between the three phases is given in the 1st column for each phase, and the second and third column give the molar and weight phase fractions.

**Figure 1:**
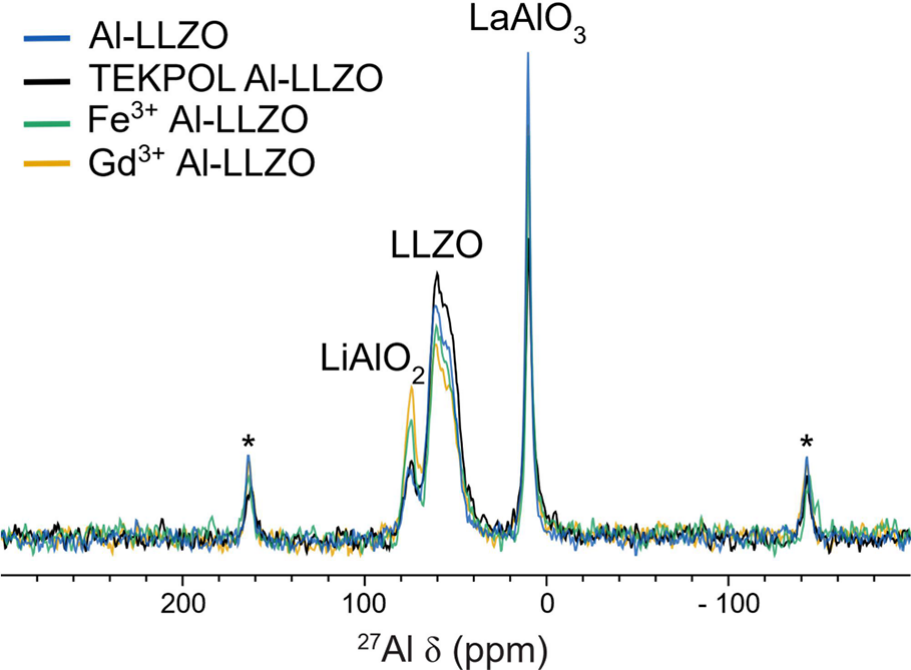
^27^Al MAS NMR spectra (11.7 T, ν_rot_ = 20 kHz, recycle delay = 1 s) of Al-LLZO and Al-LLZO with three different dopants, showing the presence of three Al environments in all Al-LLZO samples. The peaks labeled with asterisks correspond to spinning sidebands.

Paramagnetic centers are known to increase the relaxation rates of nearby nuclei,^31^ and thus, the effect and location of the paramagnetic dopants were investigated by recording the ^27^Al nuclear spin–lattice relaxation time constants (*T*_1_) of the Al-LLZO samples (Table 2). These measurements were first performed at high field (16.4 T) to better separate the resonances arising from LiAlO_2_, LaAlO_3_, and Al^3+^ in LLZO. The experiments were repeated at 100 K and at a lower field (9.4 T) for the undoped LLZO sample, to determine how the *T*_1_ times differ under the field strength and temperature used for the DNP measurements.

**Table 2:** *T*_1_ Relaxation Time Constants of ^27^Al Measured by a Saturation Recovery Experiment at Room Temperature (RT) and 100 K^a^

	Al-LLZO RT	Al-LLZO 100 K	Gd doped RT	Fe-doped RT	TEKPol RT
LiAlO_2_	14.8 s	7.0 s	7.4 s	6.3 s	14.3 s
LLZO	98.5 ms	119 ms	33.7 ms	64.9 ms	114.8 ms
LaAlO_3_	18.3 s	30.9 s	11.4 s	393.8 ms	17.9 s

a All the room temperature (RT) spectra were recorded at 700 MHz (16.4 T, ν_rot_ =15 kHz), and the 100 K data were recorded at 400 MHz (9.4 T, ν_rot_ =20 kHz).

In the undoped sample, clear differences in the *T*_1_s were observed, with the *T*_1_ of the Al^3+^ spins in LLZO being two orders of magnitude shorter than that of Al^3+^ in LaAlO_3_ and LiAlO_2_. This can be explained by the larger quadrupole coupling constant (*C*_Q_) of ^27^Al in LLZO (*C*_Q_ = 5.1 MHz, 3.0 and 0.2 MHz for LLZO, LiAlO_2_, and LiAlO_3_, respectively (Figure S2)) coupled with the presence of Li ion motion, resulting in fluctuating local magnetic fields. The Al^3+^ *T*_1_ time for LLZO lengthens somewhat at lower fields and temperatures, consistent with the slowing down of motion. However, the relaxation time is still short (119 ms), suggesting that residual motion is still present.

As expected, large changes in *T*_1_ were observed upon the addition of paramagnetic Gd^3+^ or Fe^3+^. The addition of TEKPol seems to have little influence on the relaxation times; this is expected, given that TEKPol should only cover the accessible surfaces of the particles and, hence, is only near the subset of ^27^Al nuclei that are close to the surface.

### DNP Experiments to Selectively Enhance and Identify Bulk and Impurity Phases

Having determined the relative phase fractions of the main LLZO component and impurity phases by XRD and more conventional NMR approaches, we then used a series of DNP approaches to first identify the impurities and then, finally, seek to determine where they are located. The Gd-doped samples are first investigated to identify the La-containing impurity phases. Then the effect of Gd- and Fe-doping was compared using ^27^Al DNP NMR. Finally, DNP measurements with TEKPOL are reported.

### Using Gd^3+^ as the Polarization Source: ^1^H and ^7^Li DNP NMR Spectra

For the Gd-doped sample, a full magnetic field sweep was first recorded at 100 K (Figure 2) while acquiring either the ^1^H, ^7^Li, or ^27^Al NMR spectra of Gd-doped Al-LLZO. This sweep shows positive and negative enhancements separated by twice the nuclear Larmor frequency for ^1^H, ^7^Li, and ^27^Al, proving that the DNP enhancement was achieved through the solid effect.^14^ ^1^H and ^7^Li NMR spectra (Figure 3) were then recorded by placing the static magnetic field at fields corresponding to the maximum positive enhancement of either ^1^H or ^7^Li (see Figure 2), and DNP enhancement factors of 5 and 32, respectively, were obtained when using a recycle delay, D1, of 5 s. A summary of the magnetic fields used for each dopant and nuclide is provided in the SI table (Table S2).

**Figure 2:**
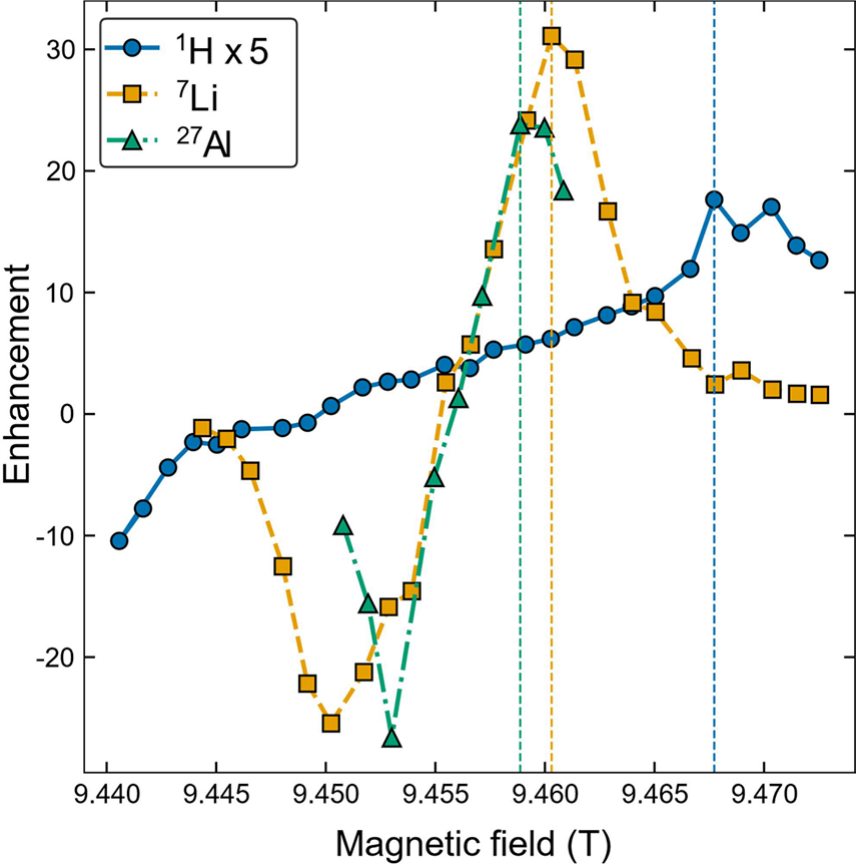
Magnetic field sweep profile of the Gd-doped Al-LLZO at 100 K showing a solid-effect type enhancement. The dashed lines indicate the field with maximum enhancements for the different nuclei and correspond to the field used for recording the DNP NMR spectra in subsequent experiments. The ^1^H enhancement is scaled up by a factor of 5 to more clearly see the maximum.

**Figure 3:**
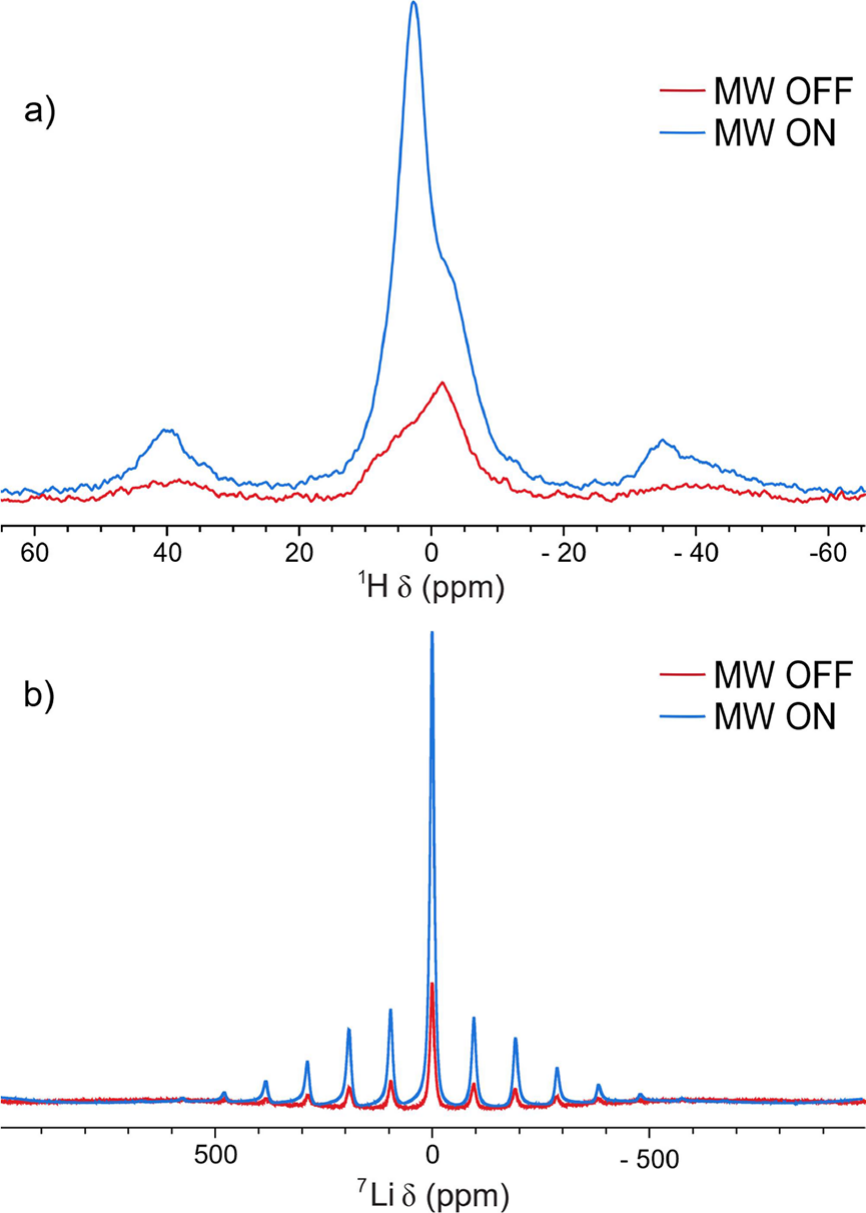
DNP MAS NMR spectra of Gd^3+^ doped LLZO (100 K). (a) ^1^H NMR spectra (9.468 T, ν_rot_ = 15 kHz, recycle delay = 5 s) showing selective enhancement of the higher frequency resonance identifying this as arising from protons in LLZO. (b) ^7^Li NMR spectra (9.460 T, ν_rot_ = 15 kHz, recycle delay = 5 s) showing enhancement but no resolution of the different lithium species. Microwave (MW) on (blue) and off (red) are shown for both sets of spectra.

While ideal LLZO does not contain any protons, any exposure to moisture or air causes surface hydroxides to form and protons to exchange for the Li ions inside the structure.^32^ The ^1^H NMR spectrum (Figure 3a) shows selective enhancement of the peak at approximately 4 ppm, indicating that this is the signal due to protons in LLZO, while the peak at around −2 ppm, which is not enhanced, is assigned to surface species such as LiOH (Figure S3). This assignment is made based on the assumption that Gd^3+^ dopes into the bulk LLZO structure and therefore selectively enhances the protons in the LLZO. This is also consistent with previous studies that used ^7^Li–^1^H correlation experiments to assign the peak at around 4 ppm to protons inside the garnet structure.^33–36^ While significant enhancement is seen in ^7^Li NMR spectra of Gd^3+^ doped LLZO (Figure 3b, and zoomed in on the centerband in Figure S4), it is not possible to determine whether any specific phase is enhanced as the ^7^Li signals of Li^+^ in LLZO and the lithium containing impurities overlap, and so it is difficult to determine which phase is associated with the largest DNP enhancement. Thus, DNP experiments were performed with other NMR-active nuclei in this system (^17^O and ^27^Al).

### Direct ^17^O DNP OF Gd^3+^-Doped LLZO

To explore Gd^3+^-doping further, some of the Gd^3+^-doped Al-LLZO was ^17^O enriched by heating the sample in ^17^O_2_ gas at 600 °C overnight. The PXRD pattern (Figure S1) showed the formation of some La_2_Zr_2_O_7_ alongside the previous LaAlO_3_ and Li_2_ZrO_3_ impurities. The ^17^O MAS NMR spectrum was recorded at high field (16.4 T) and assigned according to previous literature assignments (Figure S7). This previous work performed by some of the authors used density functional theory (DFT) calculations to estimate the chemical shifts of the different O local LLZO environments and suggested that there were at least five different local O environments with ^17^O chemical shifts ranging from 225 to 410 ppm.^37^ To investigate why the ^17^O NMR signal of LiAlO_2_ was not observed in the high-field ^17^O spectrum (Figure S7), enrichment of a pure sample of LiAlO_2_ was attempted using the same enrichment protocol. Again, no ^17^O NMR signal was observed, indicating limited-to-no ^17^O enrichment. This sample was run overnight at a high magnetic field strength (20 T) (Figure S8), and a weak resonance with a shift at around 30 ppm was observed, close to that predicted from the DFT calculations (51 ppm, Table S3).

The Gd^3+^ polarized direct ^17^O DNP spectrum was then recorded, now at a magnetic field corresponding to the maximum DNP enhancement for ^17^O (Figure 4; see Table S2 for field strength), showing enhancement of the features assigned to LLZO and the other lanthanum-containing oxide impurities present. To fit this spectrum, the microwave (MW) off-spectrum was first fitted assuming all peaks correspond to LLZO. The “on” spectrum was then fitted using the same parameters for the LLZO peaks. The La_2_Zr_2_O_7_^17^O resonance that overlaps with some of the LLZO peaks was fitted using the same line broadening parameters as the higher frequency La_2_Zr_2_O_7_ resonance. Furthermore, DFT calculations were performed to confirm the shifts and quadrupole parameters for the impurities included in these fits (Table S3). DFT calculations were also used to obtain an estimate for the ^17^O NMR shifts of LiAlO_2_ (51 ppm) and Li_2_ZrO_3_ (330 and 353 ppm), but these signals are not expected to be enhanced by the Gd^3+^ spins as they do not contain La^3+^. Furthermore, the LiAlO_2_ signal was not observed in the ^17^O spectra recorded at 16.4 T (Figure S7).

**Figure 4:**
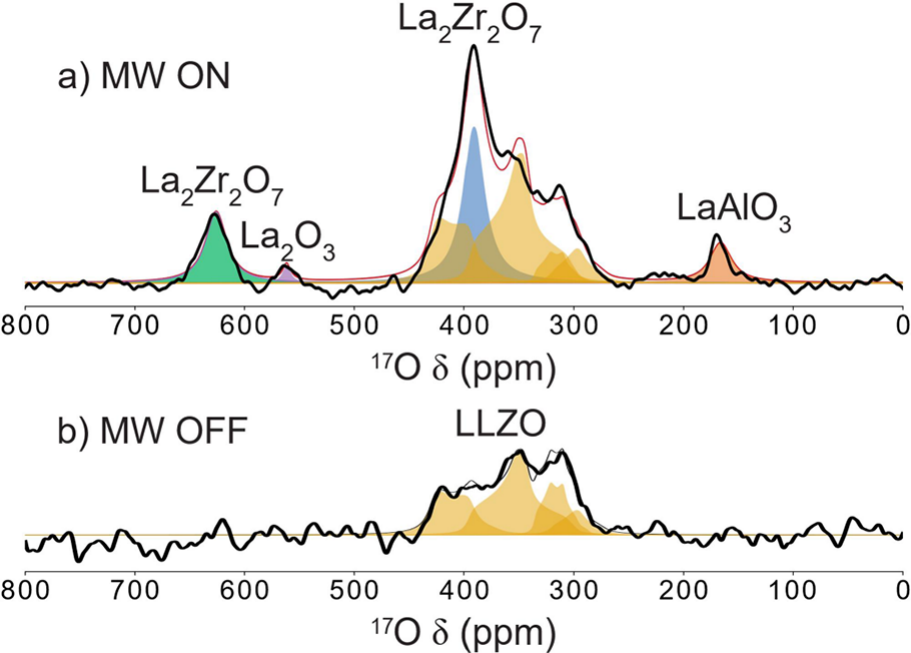
^17^O DNP NMR (9.456 T, ν_rot_ = 30 kHz, 100 K, recycle delay = 0.15 s) of ^17^O enriched Gd^3+^-doped LLZO with (a) and (b) without microwave irradiation, showing enhancement of LLZO and some of the impurity phases.

A much greater enhancement was seen for the oxygen-containing impurities than for the LLZO bulk phase. While in the MW-off spectrum it was difficult to see the peaks from LaAlO_3_, La_2_Zr_2_O_7_, and La_2_O_3_, the MW-on spectrum showed all three impurities clearly. The strong enhancements are likely due to these species being present on the surfaces of the LLZO composite, and hence, they become ^17^O-enriched more easily than LLZO. The higher enrichment levels result in, on average, shorter ^17^O–^17^O distances and hence better spin diffusion and enhancement. The lack of a LiAlO_2_ signal in the Gd^3+^-doped Al-LLZO DNP spectrum, despite this being a known impurity phase, is consistent with both unsuccessful ^17^O enrichment and no Gd^3+^ substitution in this phase.

No peaks corresponding to LiOH or Li_2_CO_3_,^38^ both common surface impurities of LLZO, were observed. This is likely due to the enrichment being performed at a temperature at which these species are removed. Finally, we note that the electronic spin–lattice relaxation time constant (*T*_1e_) of the Gd^3+^ spins is also likely different in different phases, influencing the extent of DNP enhancement.

### Direct ^27^Al DNP Using (Gd^3+^ and Fe^3+^) Endogenous Dopants

Having identified a series of impurity phases that can be doped with Gd^3+^, ^27^Al DNP experiments were performed with both endogenous doping to distinguish between doping on the La and Al sites. The magnetic field was first moved to the maximum for ^27^Al enhancement, and the ^27^Al DNP MAS NMR spectra of the Gd-doped sample were recorded, again at 100 K (Figure 5a). A weak enhancement of LLZO was seen, with the enhancement of LaAlO_3_ being much larger (factors of 2 and 26, respectively, at a recycle delay of 8 s) (see Table 3). No enhancement of the LiAlO_2_ signal was seen, consistent with the lack of Gd^3+^ doping in this phase. Note that the LiAlO_2_ signal is observable only as a weak shoulder to higher frequencies of the LLZO signal at the magnetic field strength used for the DNP experiments (9.4 T) but is much more clearly visible in higher field spectra obtained at 16.4 and 23.5 T (Figure S2). This is due to the second-order quadrupolar broadening being reduced at higher fields, which is more noticeable for the resonance assigned to Al in LLZO, given its larger associated quadrupole coupling (Figure S2).

**Figure 5:**
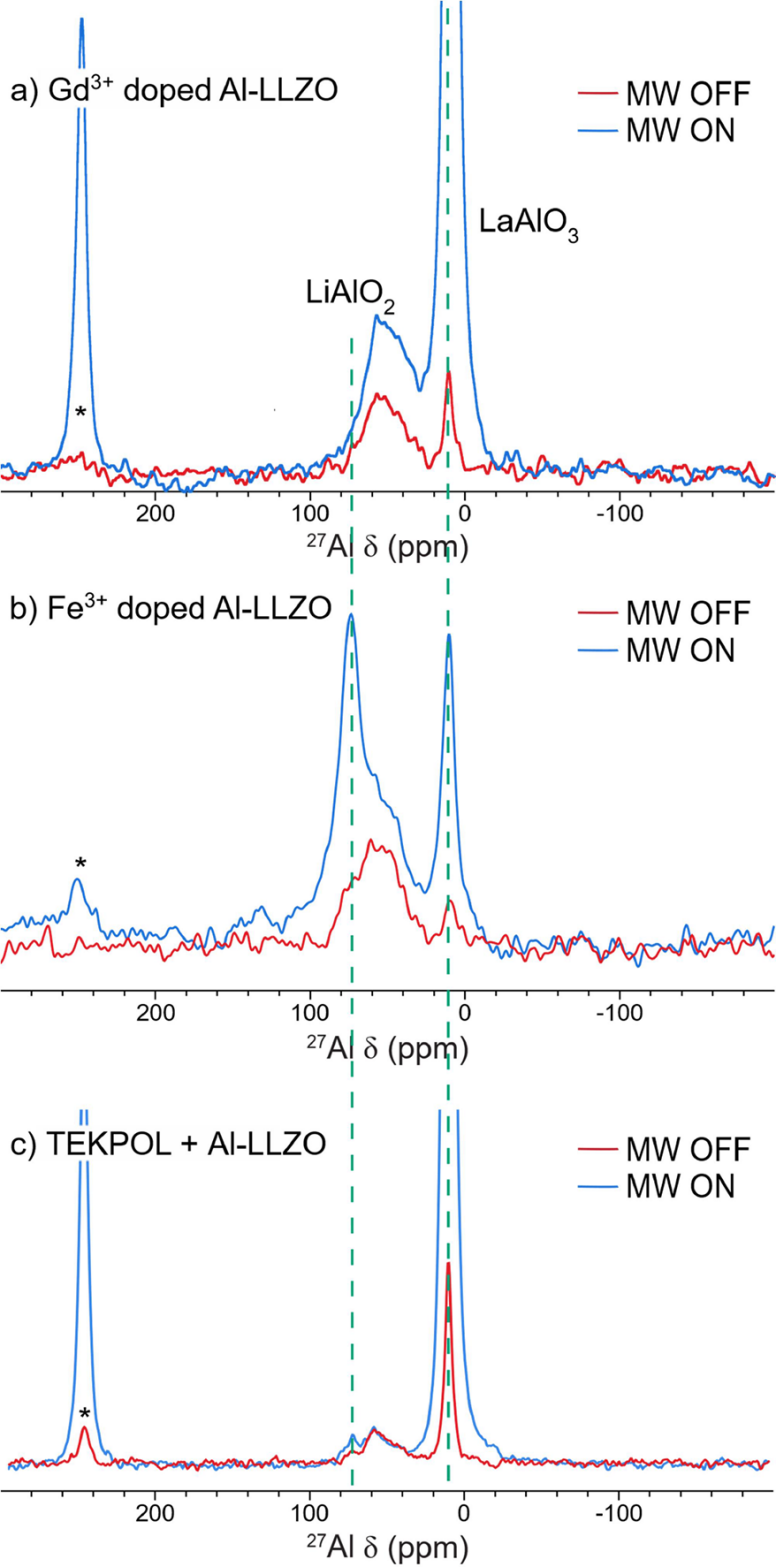
DNP MAS NMR (100 K, ν_rot_ = 25 kHz) (MW on and off) spectra of the three differently doped Al-LLZO samples. (a) Gd^3+^-doped Al-LLZO (9.459 T, recycle delay = 8 s) showing selective enhancement of LaAlO_3_. (b) Fe^3+^-doped Al-LLZO (9.403 T, recycle delay = 8 s) showing enhancement of ^27^Al in LiAlO_2_ and LaAlO_3_ indicating successful doping of Fe^3+^ into both impurities. (c) TEKPOL doped Al-LLZO (9.402 T, recycle delay = 45 s) showing significant enhancement of the LaAlO_3_ environment. The LaAlO_3_ isotropic resonance and its sideband at approximately 245 ppm have been truncated to see the enhancements of the weaker/broader signals. Longer recycle delays were used in (c) to ensure that more distant ^27^Al spins could be enhanced if effective spin-diffusion pathways were present.

**Table 3:** Summary of the ^27^Al Enhancements Seen in Direct ^27^Al DNP NMR Spectra for the LLZO, LaAlO_3_, and LiAlO_2_ Signals

polarization agent	recycle delay (s)	LLZO	LaAlO_3_	LiAlO_2_
Gd^3+^	8	2	26	0
Fe^3+^	8	1.3	9.5	8.5
TEKPol	8	0	38	insufficient S/N
TEKPol	45	0	51	2

No ^27^Al–^27^Al spin diffusion pathway was observed for Al in LLZO at RT in our previous paper,^7^ and consistent with this, the direct ^27^Al DNP experiment at room temperature (Figure S5) showed no enhancement for LLZO. The small but nonzero enhancement seen at 100 K is likely correlated with the slowing down of the Li^+^ dynamics, reducing the local fluctuations in magnetic field and increasing the ^27^Al *T*_1_, albeit only slightly. It is also likely that the Gd^3+^ *T*_1e_ increases at lower temperature, as previously reported in different compounds in the literature,^39^ increasing the achieved DNP polarization.

The Fe-doped LLZO sample was then studied. A partial field sweep was first performed, and the ^27^Al DNP NMR spectrum was recorded at the magnetic field strength corresponding to a maximum ^27^Al DNP enhancement (Figure 5b). The DNP spectrum showed enhancement of all three Al environments, with the largest enhancement being obtained for the LiAlO_2_ signal at 80.1 ppm. This was confirmed by a deconvolution of the MW-on and -off DNP spectra, which also allowed the different enhancements to be extracted (Figure S6, Table 3).

The fact that the peak at 80.1 ppm can be enhanced by the Fe^3+^ but not from Gd^3+^-spins further confirms that this peak arises from LiAlO_2_ as assigned in our previous publication.^7^ If this peak was due to Al on a site within LLZO, as suggested in the literature,^37,40^ DNP enhancements from both Gd^3+^ and Fe^3+^-doping would be expected as they both dope into the LLZO phase, albeit likely on different sites.

### Surface Polarization by TEKPol: Direct ^27^Al DNP

To determine which phases are located at or near the surface of the LLZO composite, the LLZO sample was impregnated with TEKPol, which was then used as the polarization source (Figure 5c). For the DNP to operate, a hyperfine interaction between the unpaired electrons in the radicals (i.e., TEKPol) and the nuclear spins is required to polarize the latter. In other words, the radical needs to be close to the atoms containing the nuclei of interest (here ^27^Al).

A large enhancement of LaAlO_3_ was seen (up to a factor of 51 at a recycle delay of 45 s, Table 3) when the microwave field was optimized for maximum ^27^Al enhancement (Figure S10), providing proof that a significant fraction of this impurity phase is formed or present on the surfaces of LLZO and thus accessible to the radical. Given that TEKPol is known to degrade, a new sample was prepared for each DNP session. In some samples, some enhancement of LiAlO_2_ was seen, but this was never significant (at most a factor of 2 at a recycle delay of 45 s). The lack of enhancement cannot be ascribed to poor ^27^Al–^27^Al spin diffusion in LiAlO_2_. In our previous paper,^7^ ^27^Al–^27^Al dipolar build-up curves for both the LiAlO_2_ and LaAlO_3_ impurities in LLZO were recorded at RT. Analysis of these build-up curves (see Figure S9) showed that while the ^27^Al–^27^Al dipolar coupling was stronger in LaAlO_3_ than in LiAlO_2_, both species should have effective ^27^Al spin diffusion pathways. This is further supported by the high DNP enhancement for the Fe^3+^-substituted sample, where enhancement of the LiAlO_2_ impurity was seen, again indicating an effective ^27^Al spin-diffusion mechanism. Thus, the DNP results indicate that TEKPol is, on average, closer in proximity to LaAlO_3_ than to LiAlO_2_. Again, little-to-no LLZO enhancement was seen.

### Surface Polarization by TEKPol: ^7^Li–^27^Al D-HMQC

The lack of enhancement of the ^27^Al spins in LiAlO_2_ can either be explained by it being formed in a space inaccessible to the TEKPol radical, e.g., at the LLZO grain boundaries, or by the LiAlO_2_ phases being separated from the TEKPol radical by other surface impurities. To distinguish between these two hypotheses, a DNP-enhanced ^7^Li–^27^Al double resonance experiment was performed, in which the ^7^Li spins are first polarized during the recycle delay, since ^7^Li spin diffusion in lithium-containing solids is fast^41^ and can be used to provide effective polarization of the bulk material. Furthermore, the ^7^Li spin diffusion pathway is better than the ^27^Al spin diffusion pathway, given the higher gyromagnetic ratio of ^7^Li and its smaller quadrupolar moment and nuclear spin quantum number: *I* = 3/2 vs 5/2. TEKPol was again used as the radical for DNP, and the field strength of the magnet was optimized so that the TEKPol selectively enhanced the ^7^Li and not the ^27^Al spins (Figures S10 and S11).

A ^7^Li–^27^Al DNP D-HMQC (dipolar-based heteronuclear multiple quantum correlation) experiment using rotational echo double resonance (REDOR) type recoupling (Figure 6) was chosen, since it selects for Al environments close to ^7^Li. Microwave irradiation will likely affect the signal intensities, as explored below. Given that the surfaces of LLZO are known to react to form LiOH (as seen by ^1^H NMR) and Li_2_CO_3_, even in an inert environment such as a glovebox, the polarization of the ^7^Li rather than ^27^Al spins with the TEKPol radical, may allow us to detect Al containing surface impurities even if they are covered with a thin lithium-containing surface coating.

**Figure 6:**
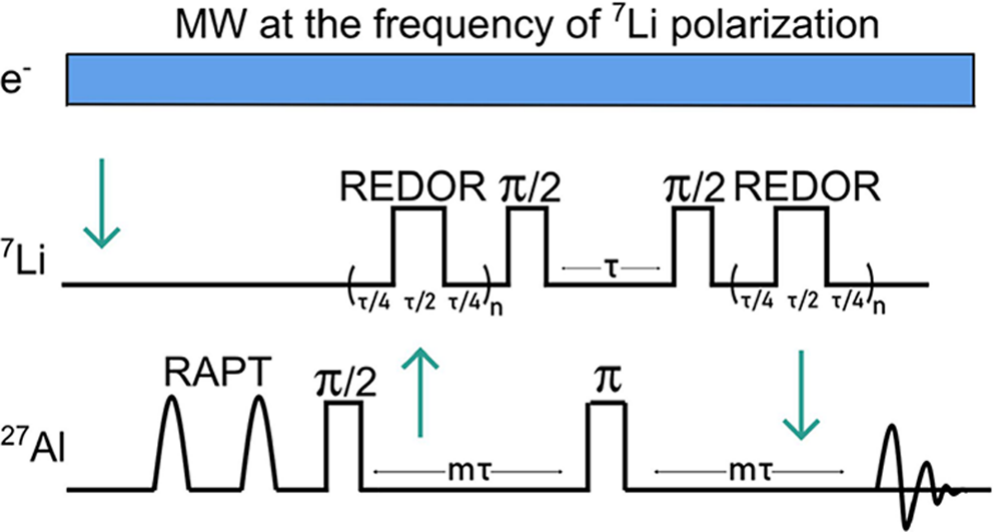
A diagram showing the ^7^Li → ^27^Al DNP D-HMQC rotor-synchronized sequence. The green arrows show the direction of the polarization transfer. A rotor assisted polarization transfer (RAPT)^42^ step was first applied to the ^27^Al satellite transitions to increase the ^27^Al central transition signal. REDOR recoupling (five rotor periods) was then applied at the ^7^Li (rather than at the ^27^Al channel) as it is difficult to achieve uniform and effective nutation of quadrupolar nuclei with moderate-to-large quadrupolar couplings such as ^27^Al, and thus the ^27^Al spin system is to poorly controlled and magnetization/coherences are rapidly lost.^24^ ^27^Al–^7^Li dipolar coupling coherence is built up, before the Al magnetization associated with this coherence is transferred to ^7^Li. Then a π pulse is applied to refocus the ^27^Al signal before returning the magnetization to the ^27^Al channel and applying the REDOR sequence to reconvert the ^27^Al–^7^Li dipolar coupling coherence into an observable signal. Appropriate phase cycling is applied so that only Al, dipolar coupled to Li, remains at the end of the sequence. One rotor period is indicated by τ.

The results of the ^7^Li–^27^Al DNP D-HMQC experiment are shown in Figure 7 as a function of the recycle delay. Both the ^27^Al peaks of LLZO and LiAlO_2_ are observed, while the LaAlO_3_ resonance is not seen, confirming that this sequence has indeed suppressed any signals that do not arise from ^27^Al–^7^Li coherences (i.e., close Li–Al proximity). Upon MW irradiation and allowing polarization build-up for longer times (via the longer recycle delay), the ^27^Al signal in LiAlO_2_ is selectively enhanced, indicating that this signal is enhanced by the DNP transfer mechanism that occurs predominantly during the recycle delay. This confirms that LiAlO_2_ is formed on the LLZO surfaces.

**Figure 7:**
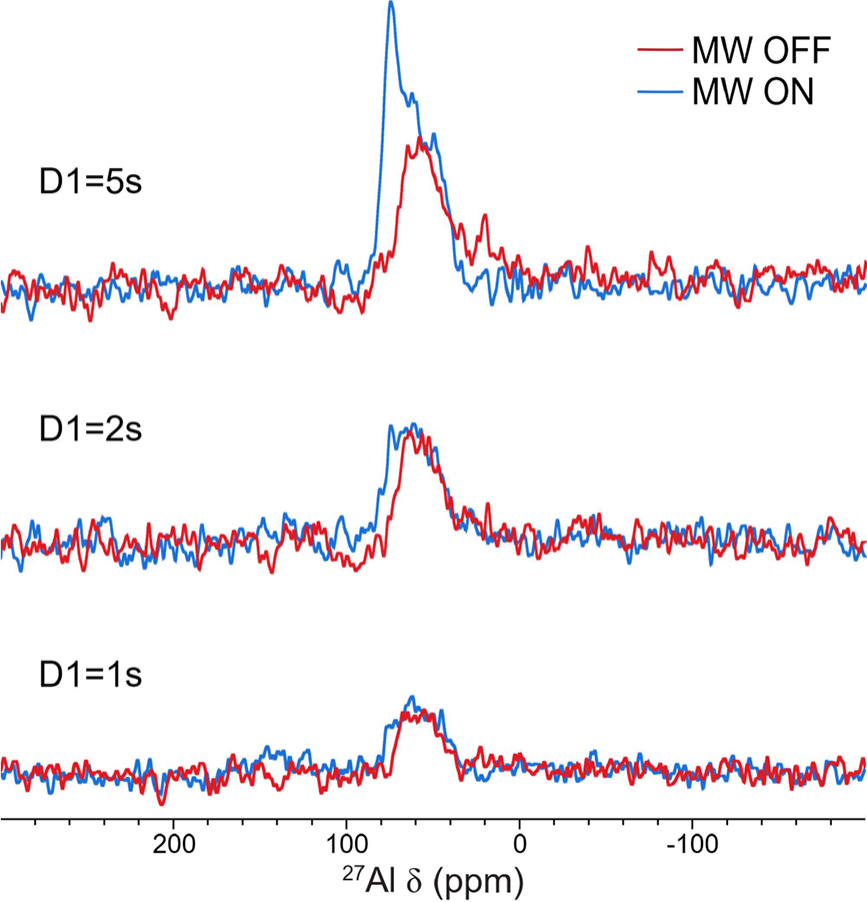
^7^Li → ^27^Al D-HMQC spectra of TEKPol doped Al-LLZO (9.398 T, ν_rot_ = 10 kHz, 100 K) run at different recycle delays (D1) at the magnetic field corresponding to maximum TEKPol DNP enhancement of ^7^Li. For these experiments, the MW off spectra were recorded by keeping the microwaves on but moving to a magnetic field where no ^7^Li or ^27^Al DNP enhancement was observed.

## Discussion

The synthesized LLZO powder forms a sintered composite (see SEM in Figure 8a) generally containing not only the desired phase, LLZO, but also a series of unwanted impurity phases, with little or no Li^+^ ionic transport. Some, but not all these phases can be detected by PXRD (see Figure S1), in part because of their low abundances, likely poor crystallinity and the presence of heavy atoms in the major phase LLZO with high scattering factors (La and to a lesser degree, Zr), which dominates the scattering. The combination of Gd^3+^ and Fe^3+^-doping and DNP, using these radicals, has clearly been shown in this work to be an effective method to enhance the signals from the impurity phases that contain sites that can accommodate these ions, providing an additional tool to examine the role of impurity phases in this important solid-state electrolyte. The ability to combine DNP with a range of different heteronuclei (here ^7^Li, ^27^Al, ^1^H, and ^17^O) allows a wider range of impurity phases and their locations of dopants to be detected and determined. To illustrate, DNP-enhanced ^1^H NMR allows Li^+^/H^+^ exchange in Gd-doped LLZO to be definitively established, with only the ^1^H signal due to the bulk phase being enhanced (Figure 3). DNP-enhanced ^17^O NMR of the enriched Gd-doped LLZO phase clearly identifies the La_2_Zr_2_O_7_ and La_2_O_3_ impurities (Figure 4). Furthermore, they are clearly found at or close to the surface of the composite, as these phases are enriched in ^17^O. A comparison of the enhancements of the ^27^Al resonances achieved via microwave irradiation of either the Gd^3+^ or Fe^3+^ spins allows substitution of Gd in the LLZO and impurity phases containing La, and Fe substitution in Al-containing phases to be confirmed (Figure 5), and the LiAlO_2_ resonance to be definitively assigned. Comparing these enhancements with those obtained when the surface radical TEKPOL is employed allows the phases that are present on the surface to be separated from those buried in the LLZO sintered composite. For example, at least some of the LaAlO_3_ is directly present at the surface of the LLZO grains. Some of the LiAlO_2_ is also found at the surface but is covered by a Li-containing film: very little direct enhancement of LiAlO_2_ is seen with TEKPOL and DNP; instead, an enhancement is only seen with TEKPOL and a DNP-enhanced D-HMQC experiment (Figure 6) where the ^7^Li magnetization is enhanced via DNP, but the ^27^Al spins are both excited and detected (Figure 7). A cartoon illustrating the LLZO composite and the location of some of the impurity phases is shown in Figure 8.

**Figure 8:**
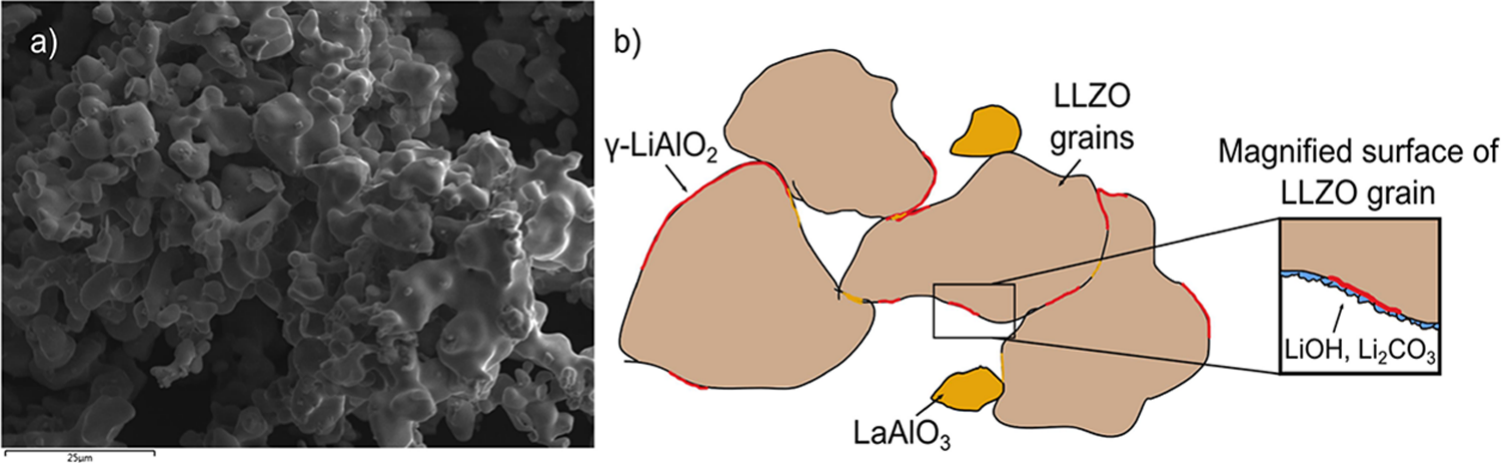
(a) SEM picture of the synthesized Al-LLZO powder. (b) Cartoon illustrating the locations of LiAlO_2_ (red) and LaAlO_3_ (yellow) impurity phases on LLZO (brown). While γ-LiAlO_2_ is primarily found on the surfaces of LLZO grains, where it is coated by phases such as LiOH and Li_2_CO_3_ (blue) or between grain boundaries, it is not directly exposed to the surface. Other impurity phases such as La_2_Zr_2_O_7_ and La_2_O_3_, which are also found near the surfaces, and that can be enriched in ^17^O when using ^17^O gas, are not shown.

The D-HMQC experiment described here has only been used in DNP experiments where the observed spin system is excited and detected, and thus, we now discuss potential mechanisms for the selective enhancement of the LiAlO_2_^27^Al signal. In general, the sensitivity observed in a D-HMQC experiment depends on the natural abundance and gyromagnetic ratio, γ_I_, of the spin that is excited and detected (*I*, here ^27^Al), and does not, to a first approximation depend on the extent of magnetization, at equilibrium, of the dipolar-coupled spin (*S*, here ^7^Li) that evolves in the indirect dimension.^43^ The fraction of *I*-spin magnetization that can then be converted into the MQ coherence (of the form *I*_*x*_ *S*_*x*_), which then evolves in the indirect dimension, depends on a number of factors including the *S*-spin natural abundance and the strength of the dipolar coupling between *I* and *S* (which depends on the gyromagnetic ratios, γ_I_ and γ_S_, and here, the Li–Al internuclear distances). When computing the evolution of the density matrix in the HMQC experiment and the evolution to produce the MQ coherences, it is generally assumed that the populations of the spins in the *S* eigenstates are approximately equal (the high temperature approximation). This is a valid approximation at low fields and ambient temperatures. However, Sugishita et al. have argued that this assumption may not be valid with the very low spin temperatures that arise from the large enhancements achieved with DNP.^44^ They calculated that with an enhancement of 1000, the spin temperatures of the ^13^C and ^1^H spins in their experiments were approximately 0.3 K, resulting in a coefficient for the two spin order (2*I*_*z*_ *S*_z_) in the density matrix that was more than 1/20 of the value of Zeeman order, *I*_Z_. They devised sequences to observe this hyperpolarization directly and, in a separate experiment, converted the dipolar order term directly into an MQ coherence and recorded a double quantum-single quantum 2D spectrum. While the enhancements obtained here are significantly lower, the breakdown of the high temperature approximation is one potential source of the additional enhancements observed for the LiAlO_2_ signal, particularly if sufficiently long relaxation times are used such that we can build up ^7^Li magnetization. It is also likely that additional terms contribute, particularly in this system where two quadrupolar nuclei are being studied, which are not effectively removed by the phase cycling, and further studies are ongoing to explore the MQ-SQ transfer mechanisms in greater detail.

More broadly, the observation that poorly crystalline γ-LiAlO_2_ is not directly exposed to the surface of the LLZO, and is either buried at the LLZO grain boundaries or covered in a coating of lithium containing salts (such as LiOH and Li_2_CO_3_) is of technological importance, because the presence of amorphous/poorly crystalline γ-LiAlO_2_, itself covered by further impurities, on the surfaces of LLZO (as illustrated schematically in Figure 8b) will hinder Li^+^ motion into the ceramic phase. This leads to current heterogeneity when LLZO is used in a solid-state battery. These observations are also consistent with the higher grain boundary resistance seen for LLZO sintered pellets that contain γ-LiAlO_2_ impurities, as compared to ones without LiAlO_2_. For example, in our previous study,^7^ we showed that the presence of γ-LiAlO_2_ can account for about 30% of total resistance in hot-pressed LLZO samples (total, grain boundary, and bulk resistivity being 2.12, 0.61, and 1.51 kΩcm, respectively). This can result in current heterogeneity near grain boundaries, which can cause additional degradation and act as soft spots for dendrite formation.^45^ However, the presence of LiAlO_2_ on the surfaces of LLZO can also help in sintering, as LiAlO_2_ is a well-known sintering agent.^46^ Consistent with this, we observed in our previous work on LLZO samples with similar Al contents that hot pressing and sintering of similar LLZO samples resulted in incorporation of the LiAlO_2_ and LaAlO_3_ impurities into the LLZO grains, as these grains grew, consistent with a well-intermixed composite. Hence, some LiAlO_2_ may be beneficial for the processing of LLZO to form dense pellets, but above a certain amount will add additional grain boundary resistances to LLZO. In addition, the LiAlO_2_, LiOH, and Li_2_CO_3_ on the surfaces of LLZO grains in as-synthesized LLZO powders will increase resistance for ion diffusion when the LLZO/polymer composite solid electrolytes are formed, and our approach may be readily extended to study these systems.

## Conclusions

This work has demonstrated a DNP-NMR approach to investigate mixed metal oxide solid electrolytes and, more specifically, the nature and locations of the impurity/grain-boundary phases formed during their synthesis. By using both endogenous and exogeneous radicals, i.e., radicals doped/substituted into the bulk or introduced onto the surfaces of the phases of interest, we were able to determine where the doping occurred and where some of the impurity phases were located. The approach was illustrated for lithium-ion conductor LLZO, whose impurity phases γ-LiAlO_2_ and LaAlO_3_ could be selectively polarized in direct ^27^Al DNP experiments by using either Fe^3+^ or Gd^3+^ dopants. This approach was also used to further confirm the identity of the Al-containing impurity phases, Gd^3+^ substituting in the La^3+^ crystalline lattice sites, selectively enhancing LaAlO_3_ while Al^3+^ substitutes into the Fe^3+^ sites, enhancing the ^27^Al signals of both γ-LiAlO_2_ and LaAlO_3_. The use of Gd^3+^ enhanced ^17^O NMR revealed a further impurity phase (La_2_O_3_), which was not seen by PXRD.

LaAlO_3_ is the only phase with a significant DNP enhancement when using direct ^27^Al DNP from TEKPol radicals dispersed on the surfaces of LLZO, indicating that it is present on the surface of LLZO rather than being completely buried in grain boundaries. However, a high enhancement of the ^27^Al signal is seen for γ-LiAlO_2_ in a ^7^Li–^27^Al D-HMQC experiment, again using a TEKPol radical. This indicates that while LiAlO_2_ and LaAlO_3_ are both present in similar quantities when synthesizing LLZO with excess Li, the LiAlO_2_ that is present at or close to the surfaces of the LLZO composite is buried underneath a film or thin layer of Li-containing impurities (such as LiOH and Li_2_CO_3_). The presence of surface films and impurity phases results in a high grain boundary resistance for sintered LLZO samples. Extensions of this NMR and DNP approach to study other complex composite materials can be readily envisaged.

## Supplementary Material



## Notes

PXRD refinements of the synthesized LLZO samples; details of the DFT calculations of ^17^O NMR parameters; list of magnetic fields at which the DNP spectra are recorded; additional NMR spectra and fit/deconvolutions of NMR/DNP spectra; ^27^Al–^27^Al dipolar build up curve of LiAlO_2_ and LaAlO_3_ in Al-LLZO; EDS of the synthesized samples; and DNP polarization build up curves and EPR (PDF)
