# Supplementary material for: Locating Impurity Phases in the Lithium-Ion Conductor Al-Doped Li7La3Zr2O12 through Dynamic Nuclear Polarization and Nuclear Magnetic Resonance Spectroscopy
Source: Chem Mater. 2025 May 13;37(10):3842–52. doi: 10.1021/acs.chemmater.5c00807 (PMC12120912; doi:10.1021/acs.chemmater.5c00807)
Supplement: Supplementary file 1 [file cm5c00807_si_001.pdf]

Locating impurity phases in the lithium-ion conductor Al-doped $Li_7La_3Zr_2O_{12}$ through dynamic nuclear polarization and nuclear magnetic resonance spectroscopy

*Astrid H. Berge, Sundeep Vema, Christopher A. O'Keefe, Clare P. Grey**

Yusuf Hamied Department of Chemistry, University of Cambridge, Lensfield Road, Cambridge CB2 1EW, U.K

SUPPORTING INFORMATION

PXRD

Samples for synchrotron PXRD were finely ground in a mortar and pestle, filled in capillaries and sealed using epoxy inside the glovebox to prevent any reaction with moisture in the air.
The capillaries were then transported to the I11 beamline at the Diamond Light Source, Oxford, United Kingdom and SXRD patterns were collected at RT in transmission mode (λ = 0.824978 Å or 0.49381 Å). The transmitted X rays were detected by position sensitive detectors.

DENSITY FUNCTIONAL THEORY CALCULATIONS

Density Functional Theory (DFT) calculations were performed using the CASTEP code.[1–13] For this, the crystal files were geometry optimized with a plane-wave basis set of 70 Ry cut-off energy and a 3x3x3 k-point grid using the PBE functional. A relativistic treatment using ZORA was added for the compounds containing lanthanum.
The NMR parameters were calculated using the same functionals and the cut-off energy and k-point grid was increased until the NMR parameters converged. This gave a convergence of 0.3ppm, 0.5ppm, 0.03, 0.01MHz and 0.0 for δ_{iso}, anisotropy, asymmetry, C_Q and η_Q respectively.

CALCULATIONS OF AL CONTENT, MOLAR FRACTIONS AND PHASE FRACTIONS

The precursors have an Al^{3+} content corresponding to a final Al-LLZO stoichiometry of $Al_{0.36}Li_{5.92}La_3Zr_2O_{12}$. If, from NMR, x % of the Al goes into LLZO, the effective LLZO formula becomes $Al_{0.36x/100}Li_{(7-1.08x)/100}La_3Zr_2O_{12}$ as each Al^{3+} replaces three Li^+.

The amount of Al per formula unit of Al-LLZO, $LiAlO_2$ and $LaAlO_3$ can then be used to quantify the molar fraction of Al-LLZO, $LiAlO_2$ and $LaAlO_3$. This is done by dividing the % Al found by NMR by the number of Al in the formula unit (0.16/0.17 in this case for Al-LLZO, and 1 for $LiAlO_2$ and $LaAlO_3$) and converting this to a percentage.

The weight fraction of Al-LLZO, $LiAlO_2$ and $LaAlO_3$ was calculated by taking the molar fraction of Al-LLZO, $LiAlO_2$ and $LaAlO_3$ and multiplying this with the molar mass of Al-LLZO, $LiAlO_2$ and $LaAlO_3$ and converting this to a percentage.

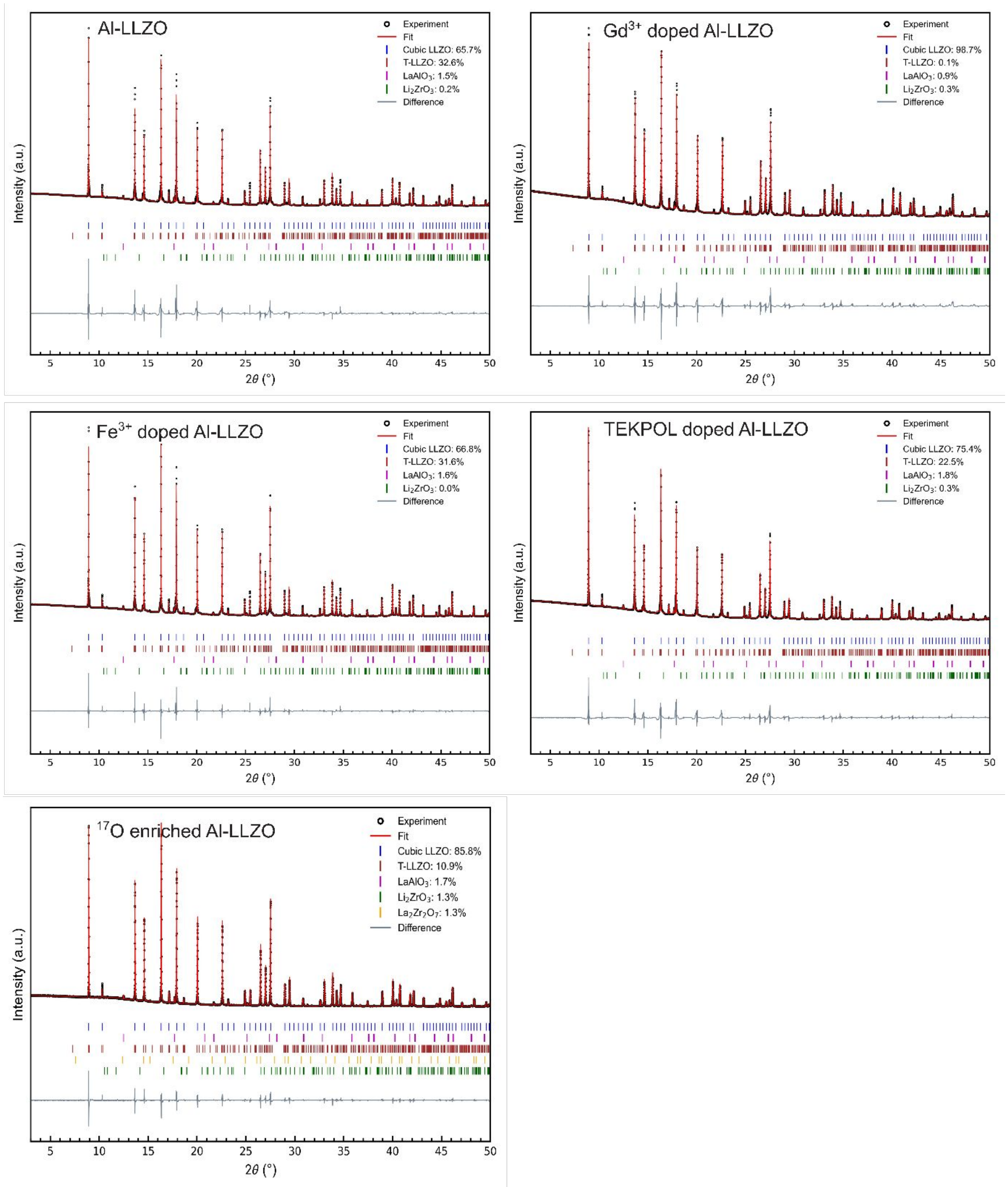

Figure S1 PXRD of the synthesized Al LLZO powders and the ^{17}O enriched Al LLZO powder. The refinements were performed using full-prof.[14]

RADII AND EXPECTED DOPANT POSITIONS

TABLE S1 Tabulated values[15] used to predict where Fe^{3+} and Gd^{3+} would dope in LLZO, $LaAlO_3$ and $LiAlO_2$.

CN	Al^{3+}		Fe^{3+} (high spin)		La^{3+}		Gd^{3+}		
	CR / Å	IR / Å	CR / Å	IR / Å	CR / Å	IR / Å	CR / Å	IR / Å	
IV	0.530	0.390	0.630	0.490					Al^{3+} in $LiAlO_2$ / LLZO
V	0.620	0.480	0.72	0.58					
VI	0.675	0.535	0.785	0.645	1.172	1.032	1.078	0.938	Al^{3+} in $LaAlO_3$
VII					1.240	1.100	1.140	1.00	
VIII			0.92	0.78	1.300	1.160	1.193	1.053	La^{3+} in LLZO
IX					1.356	1.216	1.247	1.107	
X					1.410	1.270			
XII					1.500	1.360			La^{3+} in $LaAlO_3$

MAGNETIC FIELD OVERVIEW

TABLE S2 The magnetic fields corresponding to the maximum DNP enhancement for the different DNP dopants and nuclei studied. The field of the off spectra used as comparison in the TEKPOL experiments are also noted.

	**Magnetic field (T)**					
**Sample**	**^{27}Al**	**^{7}Li**	**^{1}H**	**^{17}O**	**Off spectra ^{27}Al**	**Off spectra ^{7}Li**
**Gd doped**	9.459	9.460	9.468	9.456		
**Fe doped**	9.403					
**TEKPOL doped**	9.402	9.398			9.410	9.410

MULTIFIELD FIT OF THE ^{27}AL NMR SPECTRA OF AL-LLZO USING SSNAKE[16]

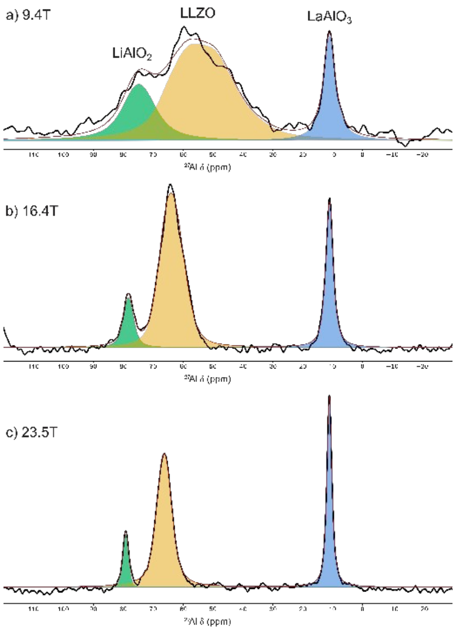

Figure S2 A simultaneous multifield fit of the NMR spectra of LLZO was preformed using data gathered at 9.4 T, 16.4 T and 23.5 T to better constrain the fitting parameters of the different Al environments. This resulted in the following parameters:

	δ_{iso} (ppm)	Cq (MHz)	η_Q
**$LiAlO_2$**	80.1	3.0	0.6
**Al-LLZO**	68.8	5.1	0.4
**$LaAlO_3$**	11.0	0.2	0.6

The fit for $LaAlO_3$ agrees well with what has been previously reported in the literature (11.7 ppm, 0.17 MHz, 0.6)[17]. Furthermore, the fits for the $LiAlO_2$ and Al-LLZO environments is consistent with that seen upon fitting an MQMAS in literature[18]

^{1}H DNP NMR OF GD^{3+} DOPED LLZO

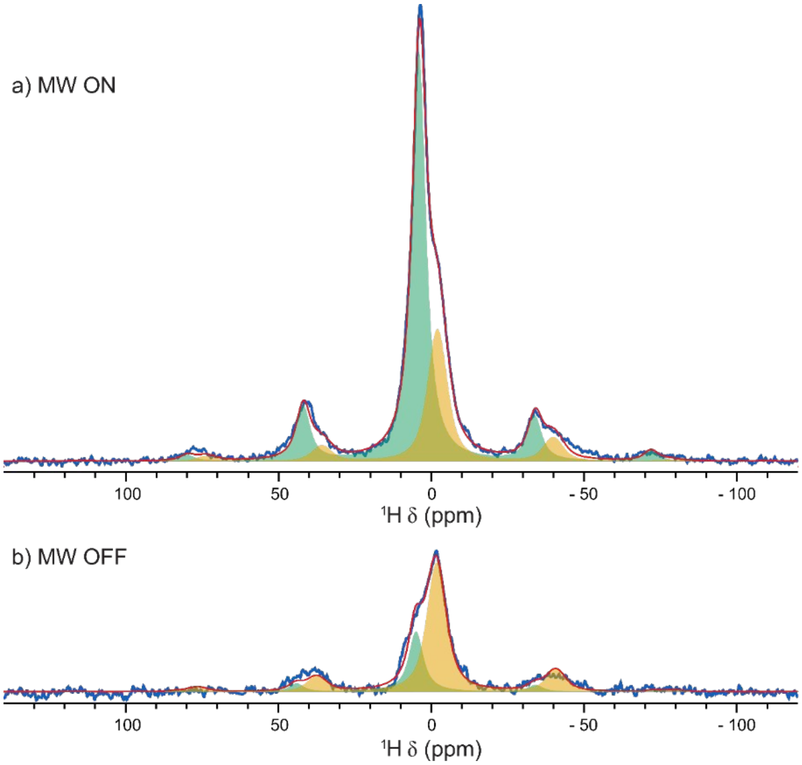

Figure S3 The ^{1}H DNP NMR spectra of Gd^{3+} doped Al LLZO (9.4 T, 15 kHz, 100K, D1=5 s) with MW irradiation (a) and without (b). The spectra were fit using SOLA and the integrals calculated using Dmfit. This gave an enhancement factor of 9.8 for the peak around 4 ppm and an enhancement factor of 1.3 for the peak at -2 ppm. The selective enhancement of the signal around 4 ppm indicates that this is the signal arising from protons within LLZO whilst the region around -2 ppm is assigned to surface impurities including protons in LiOH.

ZOOM OF ^{7}LI DNP SPECTRA OF GD3+ DOPED AL-LLZO

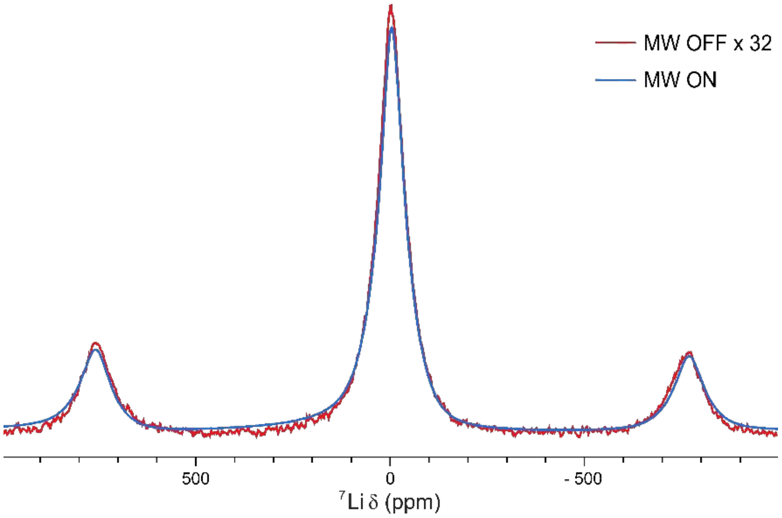

Figure S4 Magnified ^{7}Li DNP spectra of Gd^{3+} doped Al-LLZO showing the centerband and first two spinning sidebands. The off spectrum is scaled to illustrate more clearly that there is no difference in the lineshape of the off and on spectra. This means that no selective enhancement of lithium species can be seen using ^{7}Li DNP.

RT DIRECT DNP OF GD^{3+} DOPED AL-LLZO

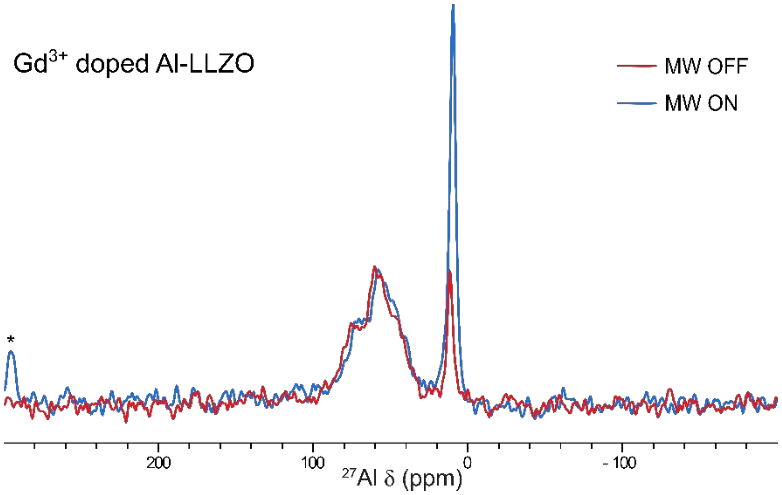

Figure S5 The RT spectra of Gd^{3+} doped Al-LLZO (9.4 T, 30 kHz, D1=1 s) showing selective enhancement of the $LaAlO_3$ peak. No enhancement of the Al in LLZO is seen.

FIT OF DNP ENHANCEMENT OF FE^{3+}

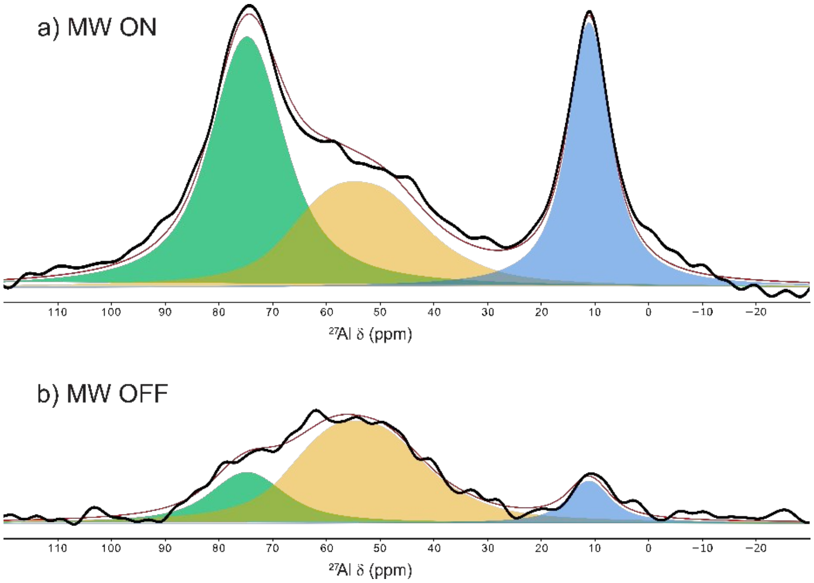

Figure S6 The ^{27}Al DNP NMR of Fe^{3+} doped LLZO (9.4 T, 25 kHz, D1=8 s) with 5.2 W MW irradiation (a) and without (b). The spectra were fit simultaneously using ssNake using the values for diso, Cq and etaQ from the previous multifield fit. Only the intensity of the peaks were allowed to vary between the spectra as the simultaneous fit was run by setting the line broadenings the same in the two spectra. The integrals of the three peaks were fitted using DMFIT and gave an enhancement of 8.9 for $LiAlO_2$, 1.3 for LLZO and 9.5 for $LaAlO_3$.

^{17}O NMR OF ^{17}O ENRICHED AL-LLZO

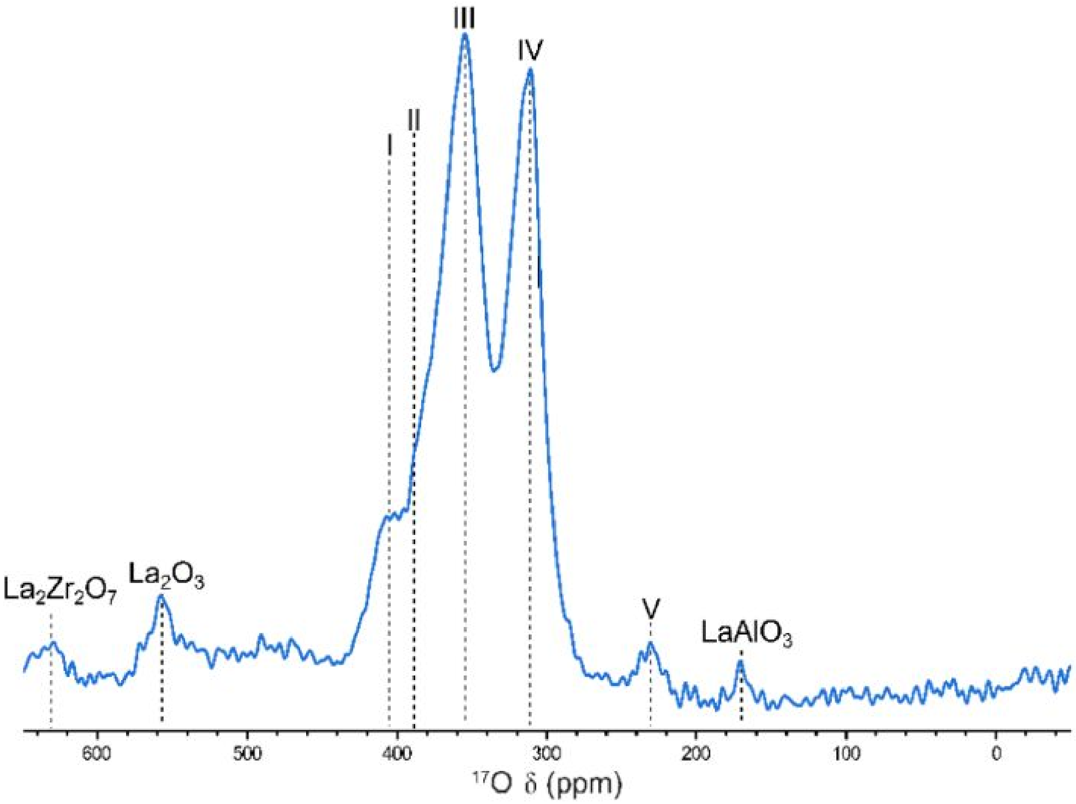

Figure S7 ^{17}O NMR spectrum (16.4 T, 50 KHz, RT) of ^{17}O enriched Al-LLZO showing the presence of several oxygen environments (I-V). The spectrum was recorded using a Hahn-echo (90-180) pulse sequence with a delay of four rotor periods.

CASTEP CALCULATION RESULTS

Table S3

^{17}O	D_{iso} (DFT)	C_Q (DFT)	N_Q (DFT)	D_{iso} (Exp)
**$LaAlO_3$**	67.9 ppm	1.54 MHz	0.26	171 ppm
**La_2O_3 site 1**	-195.2 ppm	0.89 MHz	0	470 ppm*
**La_2O_3 site 2**	-304.5 ppm	0.46 MHz	0	562 ppm
**$La_2Zr_2O_7$ site 1**	-165.5 ppm	1.09 MHz	0.96	629 ppm
**$La_2Zr_2O_7$ site 2**	-313.7 ppm	0	0	391 ppm
**$LiAlO_2$**	222.4 ppm	1.8 MHz	0.67	51 ppm*
**Li_2ZrO_3 site 1**	-55.2 ppm	0.95 MHz	0.41	330 ppm*
**Li_2ZrO_3 site 2**	-78.9 ppm	0.44 MHz	0.67	353 ppm*

*Estimated base on a calibration curve made using the other data points as not observed in recorded ^{17}O NMR. This gave a curve of D_{exp}= -D_{DFT} + 275.

^{17}O NMR OF LIALO$_2$

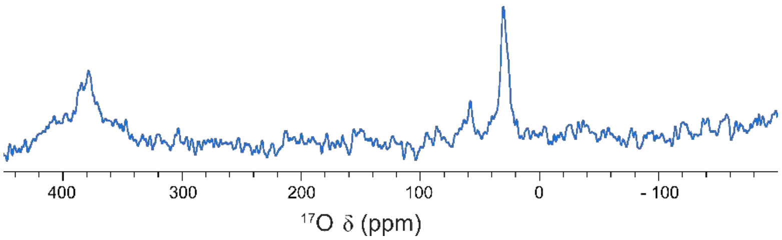

Figure S8 ^{17}O NMR of $LiAlO_2$ recorded using a one pulse sequence (20.0 T, 40 kHz, D1=0.15 s). This sample was ^{17}O-enriched using the same conditions as that of LLZO (600 °C, overnight). The resultant spectrum showed two features, one around 380 ppm corresponding to ZrO_2 and another around 30 ppm assigned to $LiAlO_2$. This assignment fits reasonably well with the expected shift of ^{17}O in $LiAlO_2$ as calculated by DFT (Table S3). Given that the intensity of the $LiAlO_2$ is of the order of that of the ZrO_2 rotor, no ^{17}O enrichment of $LiAlO_2$ has occurred at this temperature.

THE ^{27}AL – ^{27}AL DIPOLAR BUILD UP CURVES BASED ON A BR2$\frac{1}{2}$ RECOUPLING SEQUENCE AT RT.

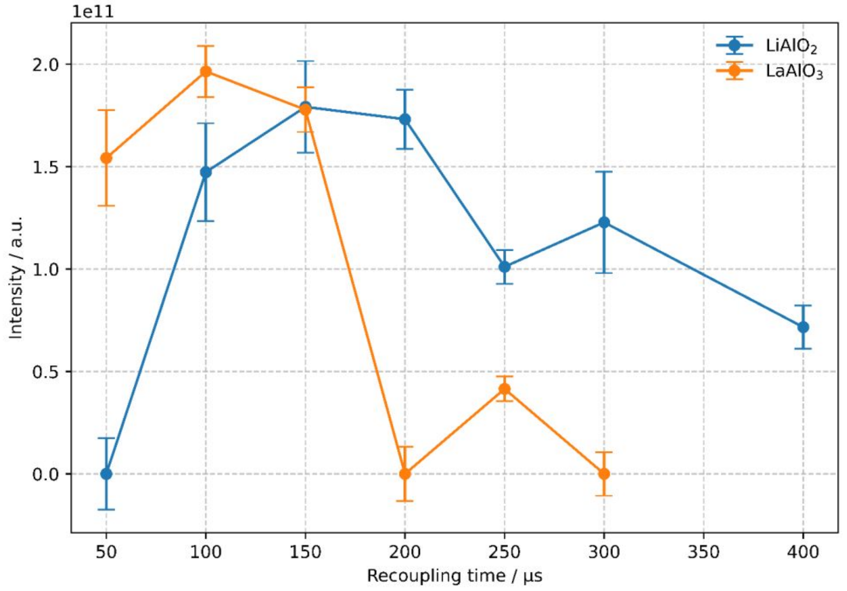

Figure S9 The ^{27}Al – ^{27}Al dipolar build up curve for $LiAlO_3$ and $LaAlO_3$ peaks in Al-LLZO (23.5 T, 40 kHz, RT)

FIELD SWEEP PROFILE OF TEKPOL DOPED LLZO

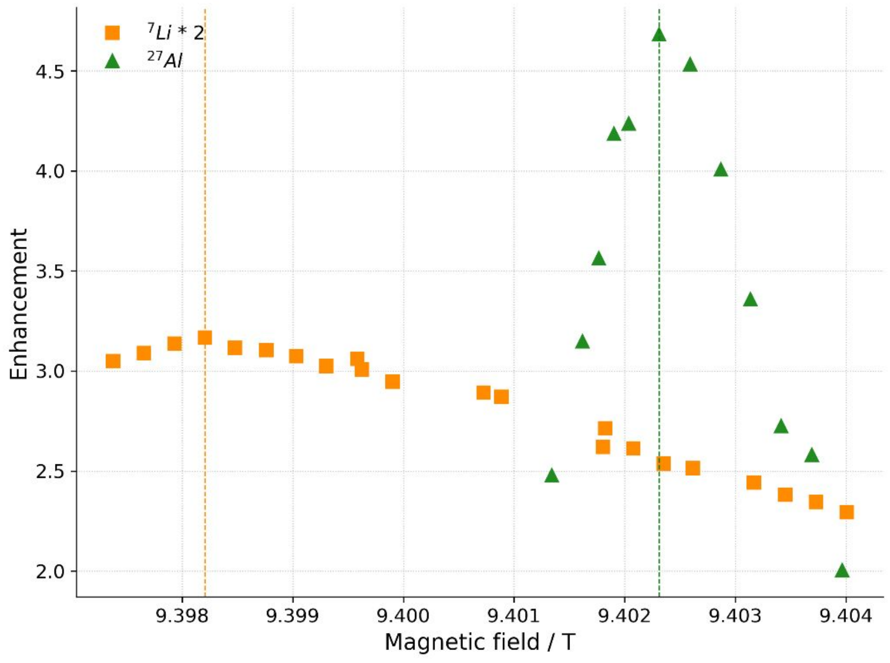

Figure S10 The DNP enhancement of ^{7}Li and ^{27}Al was recorded for the TEKPOL doped sample as the magnetic field was swept to identify the fields with the maximum enhancements. The orange dashed line indicate the field used for direct ^{7}Li DNP experiments and the ^{7}Li – ^{27}Al D-HMQC experiment. The green dashed line shows the field used for the direct ^{27}Al DNP experiments.

^{27}AL DNP AT ^{7}LI AND ^{27}AL MAXIMUM ENHANCEMENT FIELD

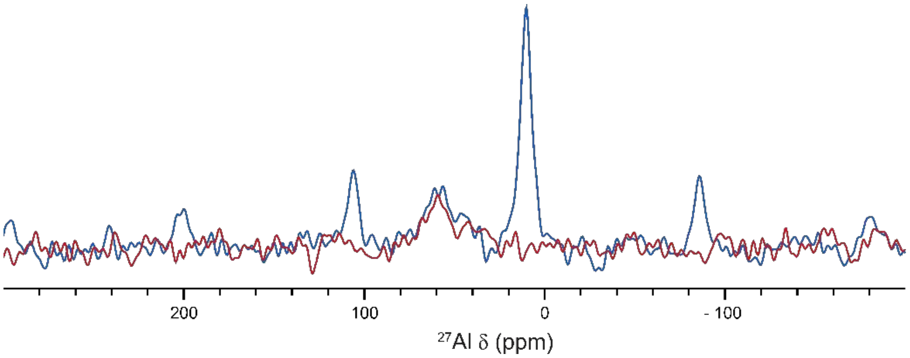

Figure S11 The ^{27}Al DNP NMR spectra (10 kHz, 100K) recorded at the max ^{27}Al DNP enhancement (blue, 9.402 T) and max ^{7}Li DNP enhancement (red, 9.398 T). This shows that there is no direct ^{27}Al polarization in the D-HMQC experiment.

SEM-EDS

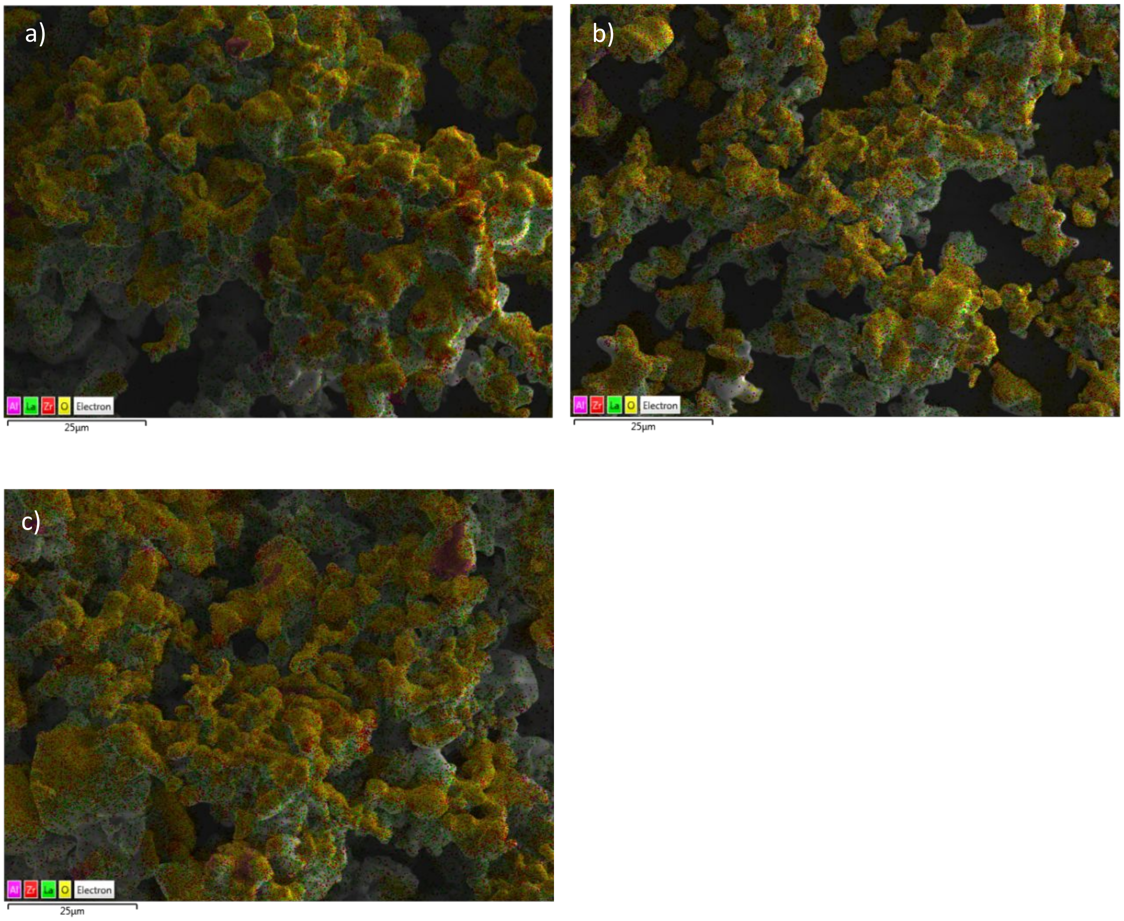

Figure S12 SEM-EDS of a) Al-LLZO, b) Fe doped Al-LLZO and c) Gd doped Al-LLZO recorded on an Oxford Instruments X-maxN 80 EDS system.

POLARISATION BUILD UP CURVES

The polarisation build-up curve of ^{27}Al were measured using saturation recovery experiments. The experiment was run with and without MW irradiation of 5.2 W to see the DNP effect.

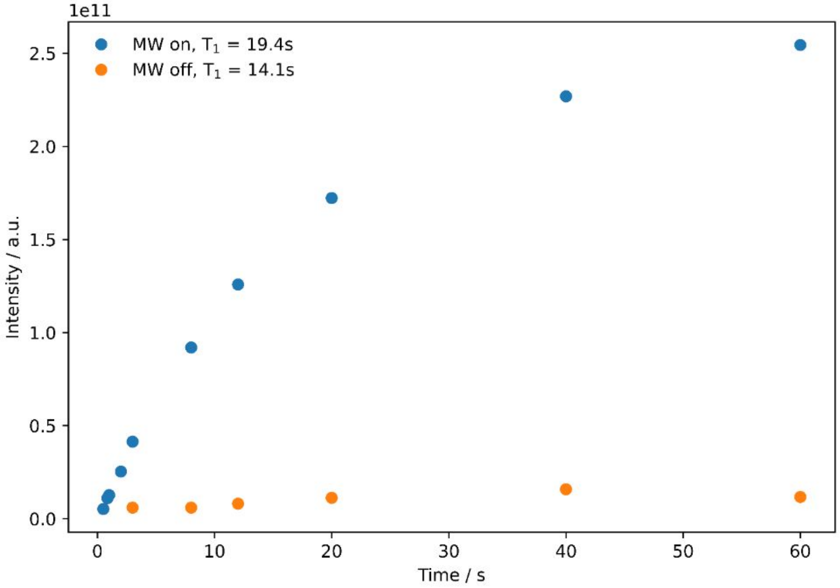

Figure S13 Polarisation build-up of $LaAlO_3$ peak in Gd^{3+} doped Al-LLZO. T1 ON = 19.4 s, T1 OFF=14.1 s

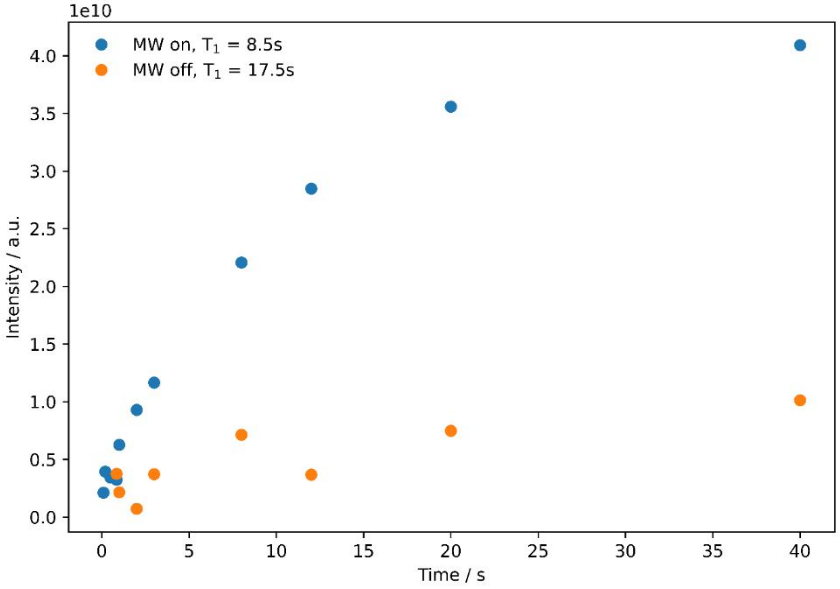

Figure S14 Polarisation build-up of $LiAlO_2$ peak in Fe^{3+} doped Al-LLZO

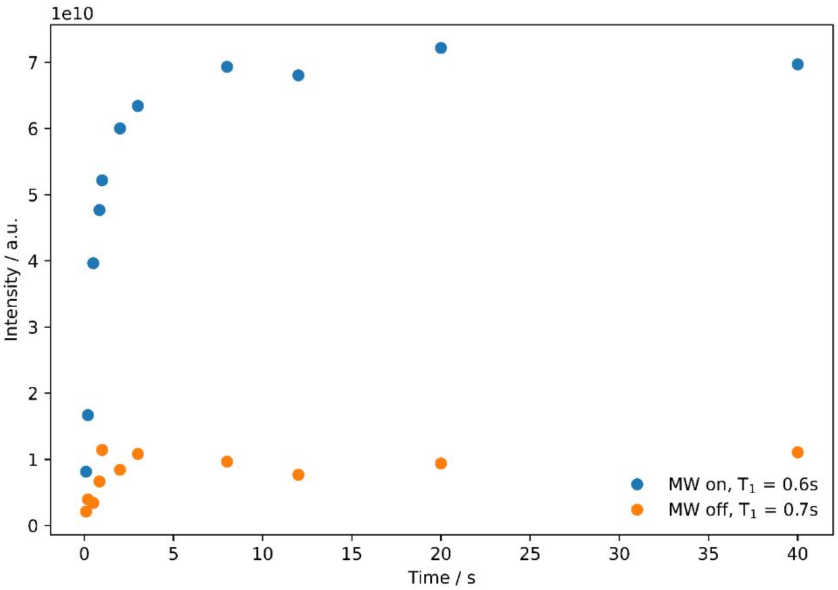

Figure S15 Polarisation build-up of $LaAlO_3$ peak in Fe^{3+} doped Al-LLZO

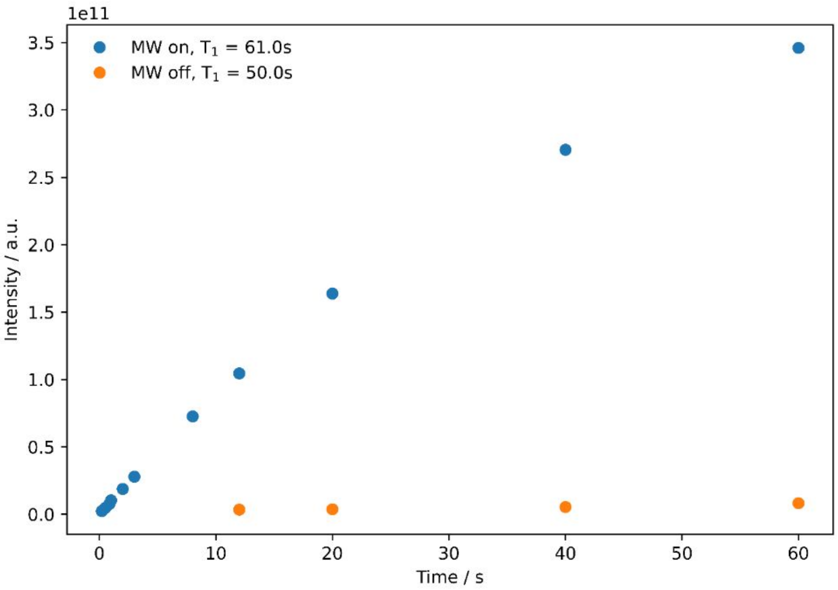

Figure S16 Polarisation build-up of $LaAlO_3$ peak in TEKPOL doped Al-LLZO

EPR OF GD^{3+}AND FE^{3+} DOPED AL-LLZO

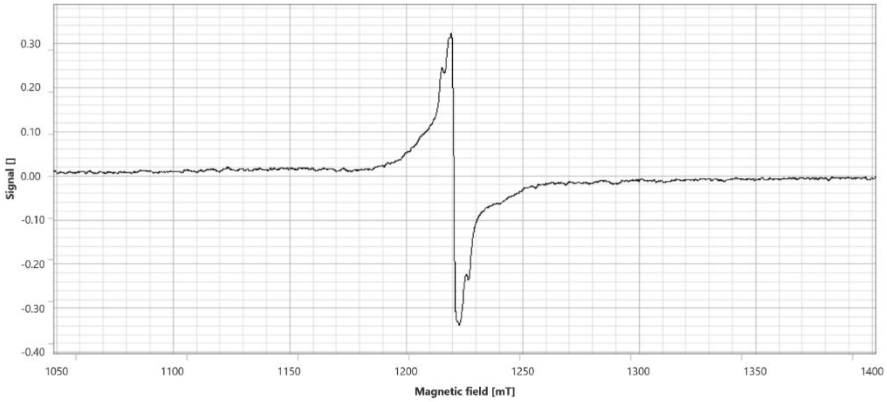

Figure S17 Q-band EPR of Gd doped Al-LLZO taken at 100K. Recorded with 5G modulation amplitude and 100kHz modulation frequency.

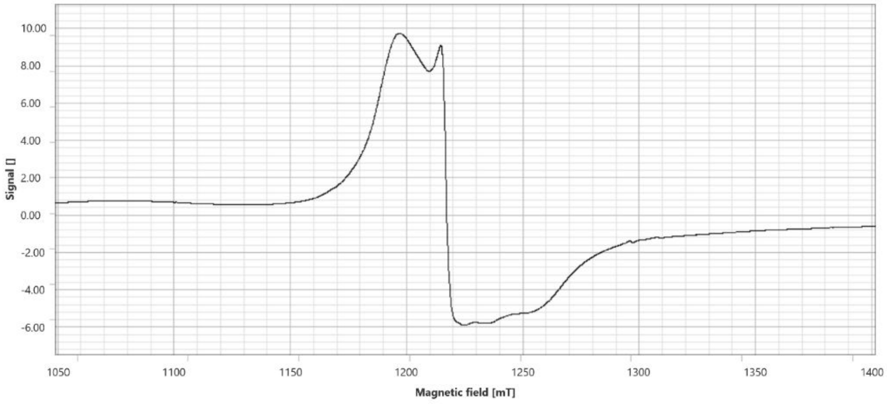

Figure S18 Q-band EPR of Fe doped LLZO taken at 100 K. Recorded with 5 G modulation amplitude and 100 kHz modulation frequency.

REFERENCES

(1) Hohenberg, P.; Kohn, W. Inhomogeneous Electron Gas. *Phys. Rev.* **1964**, *136* (3B), B864–B871. https://doi.org/10.1103/PhysRev.136.B864.

(2) Kohn, W.; Sham, L. J. Self-Consistent Equations Including Exchange and Correlation Effects. *Phys. Rev.* **1965**, *140* (4A), A1133–A1138. https://doi.org/10.1103/PhysRev.140.A1133.

(3) Monkhorst, H. J.; Pack, J. D. Special Points for Brillouin-Zone Integrations. *Phys. Rev. B* **1976**, *13* (12), 5188–5192. https://doi.org/10.1103/PhysRevB.13.5188.

(4) Payne, M. C.; Teter, M. P.; Allan, D. C.; Arias, T. A.; Joannopoulos, J. D. Iterative Minimization Techniques for Ab Initio Total-Energy Calculations: Molecular Dynamics and Conjugate Gradients. *Rev. Mod. Phys.* **1992**, *64* (4), 1045–1097. https://doi.org/10.1103/RevModPhys.64.1045.

(5) Clark, S. J.; Segall, M. D.; Pickard, C. J.; Hasnip, P. J.; Probert, M. I. J.; Refson, K.; Payne, M. C. First Principles Methods Using CASTEP. *Zeitschrift für Kristallographie - Crystalline Materials* **2005**, *220* (5–6), 567–570. https://doi.org/10.1524/zkri.220.5.567.65075.

(6) Perdew, J. P.; Burke, K.; Ernzerhof, M. Generalized Gradient Approximation Made Simple. *Phys. Rev. Lett.* **1996**, *77* (18), 3865–3868. https://doi.org/10.1103/PhysRevLett.77.3865.

(7) Pickard, C. J.; Mauri, F. All-Electron Magnetic Response with Pseudopotentials: NMR Chemical Shifts. *Phys. Rev. B* **2001**, *63* (24), 245101. https://doi.org/10.1103/PhysRevB.63.245101.

(8) Yates, J. R.; Pickard, C. J.; Mauri, F. Calculation of NMR Chemical Shifts for Extended Systems Using Ultrasoft Pseudopotentials. *Phys. Rev. B* **2007**, *76* (2), 024401. https://doi.org/10.1103/PhysRevB.76.024401.

(9) Profeta, M.; Mauri, F.; Pickard, C. J. Accurate First Principles Prediction of 17O NMR Parameters in SiO2: Assignment of the Zeolite Ferrierite Spectrum. *J. Am. Chem. Soc.* **2003**, *125* (2), 541–548. https://doi.org/10.1021/ja027124r.

(10) Bonhomme, C.; Gervais, C.; Babonneau, F.; Coelho, C.; Pourpoint, F.; Azaïs, T.; Ashbrook, S. E.; Griffin, J. M.; Yates, J. R.; Mauri, F.; Pickard, C. J. First-Principles Calculation of NMR Parameters Using the Gauge Including Projector Augmented Wave Method: A Chemist's Point of View. *Chem. Rev.* **2012**, *112* (11), 5733–5779. https://doi.org/10.1021/cr300108a.

(11) Francis, G. P.; Payne, M. C. Finite Basis Set Corrections to Total Energy Pseudopotential Calculations. *J. Phys.: Condens. Matter* **1990**, *2* (19), 4395. https://doi.org/10.1088/0953-8984/2/19/007.

(12) *Relaxation of Crystals with the Quasi-Newton Method - ScienceDirect.* https://www.sciencedirect.com/science/article/abs/pii/S0021999196956120 (accessed 2025-01-02).

(13) Byrd, R. H.; Nocedal, J.; Schnabel, R. B. Representations of Quasi-Newton Matrices and Their Use in Limited Memory Methods. *Math. Program.* **1994**, *63* (1–3), 129–156.

(14) Rodríguez-Carvajal, J. Recent Advances in Magnetic Structure Determination by Neutron Powder Diffraction. *Physica B: Condensed Matter* **1993**, *192* (1), 55–69. https://doi.org/10.1016/0921-4526(93)90108-I.

(15) Shannon, R. D. Revised Effective Ionic Radii and Systematic Studies of Interatomic Distances in Halides and Chalcogenides. *Acta Cryst A* **1976**, *32* (5), 751–767. https://doi.org/10.1107/S0567739476001551.

(16) van Meerten, S. G. J.; Franssen, W. M. J.; Kentgens, A. P. M. ssNake: A Cross-Platform Open-Source NMR Data Processing and Fitting Application. *Journal of Magnetic Resonance* **2019**, *301*, 56–66. https://doi.org/10.1016/j.jmr.2019.02.006.

(17) Blanc, F.; Middlemiss, D. S.; Buannic, L.; Palumbo, J. L.; Farnan, I.; Grey, C. P. Thermal Phase Transformations in LaGaO3 and LaAlO3 Perovskites: An Experimental and Computational Solid-State NMR Study. *Solid State Nuclear Magnetic Resonance* **2012**, *42*, 87–97. https://doi.org/10.1016/j.ssnmr.2012.01.003.

(18) Karasulu, B.; Emge, S. P.; Groh, M. F.; Grey, C. P.; Morris, A. J. Al/Ga-Doped Li7La3Zr2O12 Garnets as Li-Ion Solid-State Battery Electrolytes: Atomistic Insights into Local Coordination Environments and Their Influence on 17O, 27Al, and 71Ga NMR Spectra. *J. Am. Chem. Soc.* **2020**, *142* (6), 3132–3148. https://doi.org/10.1021/jacs.9b12685.
